# Multistability and Reversibility of Aerobic Granular Sludge Microbial Communities Upon Changes From Simple to Complex Synthetic Wastewater and Back

**DOI:** 10.3389/fmicb.2020.574361

**Published:** 2020-11-26

**Authors:** Aline Adler, Christof Holliger

**Affiliations:** Laboratory for Environmental Biotechnology, School for Architecture, Civil and Environmental Engineering, Environmental Engineering Institute, Ecole Polytechnique Fédérale de Lausanne, Lausanne, Switzerland

**Keywords:** bacterial communities, multistability, functional redundancy, PAO, *Candidatus* Accumulibacter, *Tetrasphaera*, polymeric substrates

## Abstract

Aerobic granular sludge (AGS) is a promising alternative wastewater treatment to the conventional activated sludge system allowing space and energy saving. Basic understanding of AGS has mainly been obtained using simple wastewater containing acetate and propionate as carbon source. Yet, the aspect and performances of AGS grown in such model systems are different from those obtained in reactor treating real wastewater. The impact of fermentable and hydrolyzable compounds on already formed AGS was assessed separately by changing the composition of the influent from simple wastewater containing volatile fatty acids to complex monomeric wastewater containing amino acids and glucose, and then to complex polymeric wastewater containing also starch and peptone. The reversibility of the observed changes was assessed by changing the composition of the wastewater from complex monomeric back to simple. The introduction of fermentable compounds in the influent left the settling properties and nutrient removal performance unchanged, but had a significant impact on the bacterial community. The proportion of Gammaproteobacteria diminished to the benefit of Actinobacteria and the Saccharibateria phylum. On the other hand, the introduction of polymeric compounds altered the settling properties and denitrification efficiency, but induced smaller changes in the bacterial community. The changes induced by the wastewater transition were only partly reversed. Seven distinct stables states of the bacterial community were detected during the 921 days of experiment, four of them observed with the complex monomeric wastewater. The transitions between these states were not only caused by wastewater changes but also by operation failures and other incidences. However, the nutrient removal performance and settling properties of the AGS were globally maintained due to the functional redundancy of its bacterial community.

## 1. Introduction

Aerobic granular sludge (AGS) technology is an energy-efficient and low-footprint requiring wastewater treatment process. AGS is obtained from an enhanced biological phosphorus removal (EBPR) activated sludge inoculum by applying a combination of conditions known to favor granulation while maintaining good biological nutrient performance (e.g., alternance of anaerobic feast and aerobic famine phases, high shear stress and short settling time) (Morgenroth et al., 1997; Beun et al., 1999; Barr et al., 2016). Yet it is believed that the process can be improved further by understanding the function and interactions of the microorganisms composing AGS and adapting the operational conditions consequently (Pronk et al., 2015b).

Research on AGS was, and still is, often performed in lab-scale reactors with simple monomeric synthetic wastewater containing volatile fatty acids (VFA), such as acetate and propionate as carbon source (Beun et al., 1999; de Kreuk et al., 2005, 2007; Bin et al., 2015; Barr et al., 2016; Sarma et al., 2017; Zhou and Sun, 2020). Substantial knowledge has been obtained from those studies. Importantly, it documented the negative impact of COD that was not consumed during the anaerobic phase and leaks in the aerobic phase, on granulation and nutrient removal performance (Weissbrodt et al., 2014). Nevertheless, in real wastewater, generally more than 50% of the chemical oxygen demand (COD) is present in the form of polymeric substances that cannot be readily taken up by bacteria before undergoing hydrolysis (Levine et al., 1991; Koch et al., 2000; Nielsen et al., 2010) and have a limited diffusibility across biofilms (Drury et al., 1993; Janning et al., 1998). The particulate COD in the influent wastewater is therefore likely to leak in the aerobic phase (de Kreuk et al., 2010), thus compromising the granulation and nutrient removal performance of the AGS (Bassin et al., 2012; Wagner et al., 2015). In consequence, the knowledge acquired on AGS treating simple monomeric wastewater cannot be simply transposed to AGS treating more complex wastewater containing polymeric compounds (Wagner et al., 2015). However, to date, only a minority of studies on AGS are performed with wastewater containing substantial proportions of polymeric compounds (Dulekgurgen et al., 2003; Lin et al., 2003; Schwarzenbeck et al., 2004; de Kreuk and van Loosdrecht, 2006; Lemaire et al., 2008; de Kreuk et al., 2010; Layer et al., 2019). Big differences in the physical characteristics (size, density), the settling properties and the nutrient removal efficiency of AGS grown in lab-scale reactors treating simple monomeric wastewater and full-scale tank treating real wastewater have been reported (Winkler et al., 2013; Wagner et al., 2015; Wang et al., 2018; Layer et al., 2019). Yet it is not simple to untangle the effects of the different variables on the AGS characteristics. It can be expected that the bacterial communities of these AGS differ from the ones of AGS treating simple wastewater, which contain mainly classical phosphate-accumulating organisms (PAO) and glycogen-accumulating organisms (GAO) from the class Gammaproteobacteria known to favor granulation and good nutrient removal (de Kreuk and van Loosdrecht, 2004). To date, only few studies investigated the microbial communities of AGS treating complex wastewater (Cetin et al., 2018; Kang et al., 2018; Swiatczak and Cydzik-Kwiatkowska, 2018; Wang et al., 2018; Layer et al., 2019; Dasgupta et al., 2020), and the influence of fermentable and polymeric compounds has, to our knowledge, not been studied separately.

From an engineer's point of view, it is important to assess the functional resistance of AGS microbial communities, which is the level to which the function of this community remains constant when facing a perturbation (Botton et al., 2006; Allison and Martiny, 2008). It is therefore crucial to understand the link between microbial and functional stability, how a microbial community changes in response to variations in an environmental parameter, and how these changes impact the global function of the community. The functional stability of a microbial ecosystem, which is the ability to recover a state close to its former equilibrium state after a temporary perturbation (Holling, 1973; Walter, 1980), often correlates with its biodiversity (Siripong and Rittmann, 2007; Wittebolle et al., 2008, 2009). More specifically, this stability is thought to be mainly the result of functional diversity (Hulot et al., 2000; McCann, 2000; von Canstein et al., 2002; Briones and Raskin, 2003). Yet, the functional redundancy of a microbial community is often difficult to measure because the metabolism(s) of an important part of a microbial ecosystem is generally unknown and often difficult to assess (Botton et al., 2006; Allison and Martiny, 2008). In AGS, several key functional groups are crucial for good nutrient removal and settling properties. Fermenters (e.g., *Tetrasphaera, Streptococcus*) and hydrolyzers (e.g., *Curvibacter*) play a crucial role in COD removal, ammonium-oxidizing bacteria (e.g., *Nitrosospira, Nitrosomonas*), nitrite-oxidizing bacteria (e.g., *Nitrospira*), and denitrifiers (e.g., *Thauera, Azoarcus, Zoogloea*) participate in total nitrogen (TN) removal and PAO [e.g., *Candidatus* (*Ca*.) Accumulibacter, *Tetrasphaera*] are responsible for most of the P-removal (Nielsen et al., 2010). Yet the composition of the influent wastewater in terms of carbon source can impact the structure of these functional groups.

AGS treating complex polymeric wastewater generally contains a non-negligible proportion of flocs (around 20%) (Pronk et al., 2015b; Derlon et al., 2016; van Dijk et al., 2018). Flocs are likely playing an important role in polymeric COD removal due to different physical structures providing different capabilities to capture particles (Liu and Tay, 2012). So far, no clear consensus exists on the difference of microbial communities between granules and flocs.

The experiments presented in the current article investigated separately the impact of fermentable monomeric and polymeric compounds on the bacterial communities and the function of AGS adapted to simple monomeric wastewater containing acetate and propionate only. The changes in wastewater composition were performed progressively over several weeks in order to promote at the most the maintenance of granulation and nutrient removal capability of the sludge. The effect of these transitions on the AGS and their reversibility were investigated by monitoring the bacterial community composition, the nutrient removal performance and the sludge settling properties. Statistical analysis was used to investigate the functional stability of the AGS in relation with the microbial communities.

## 2. Materials and Methods

### 2.1. Reactors Set-Up and Operation

The AGS was studied in bubble column sequencing batch reactors (SBR) with a diameter of 6.2 cm and a working volume of 2.4 L similar to the ones described in Weissbrodt et al. (2012) and Lochmatter and Holliger (2014). The sludge retention time was maintained between 20 and 30 days. The temperature was kept constant by recirculation of 18°C water in the double wall of the reactor. The pH of the bulk water was monitored and regulated with an ISFET probe (ENdress + Hauser, Switzerland) using a PID process controlling the injection of NaOH or HCl solutions (1%) during the periods of mixing. The pH was maintained between 6.5 and 8 from day 0 to 119 and between 7.2 and 8.5 from day 120 onward. The pO_2_ and the conductivity were monitored by two ISFET probes (Endress + Hauser, Switzerland). The monitoring of information collected by the reactor probes and the control of the different pumps was relayed to/from a computer through relay modules (WAGO, Switzerland) and processed using the software DAQFactory (Windows version 5.86, 2011, AzeoTech, Inc.).

A typical cycle consisted of four phases: an anaerobic feeding phase, an aerobic phase, a settling and a withdrawal phase. During the anaerobic phase, the synthetic wastewater was injected from the bottom during 12 min. Then the reactor was mixed by injecting 2 l/min of N_2_ during 60–90 min. During the aerobic phase, compressed air was injected with a flow rate of 2 l/min. Low simultaneous nitrification-denitrification was detected in our system, therefore, from day 48 on, periods of 10 min of aeration were alternated with idle periods of 10 min in order to optimize denitrification (Lochmatter et al., 2014). The aerobic phase stopped when a sharp decrease of O_2_ consumption was detected, with a maximum time of 5 h. Half of the reactor content was withdrawn after 2–5 min of settling and N_2_ was injected in the reactor to restore anaerobic/anoxic conditions for the following cycle.

The length of the different phases were adapted to allow an optimal functioning of the AGS reactors. For example, the length of the anaerobic phase was increased from 72 up to 102 min mostly during changes of wastewater composition in order to target a COD in the bulk water below 20 mg O_2_/L before start of aeration. The settling time was adapted to the settling properties of the biomass in order to select fast settling granules while maintaining sufficient biomass in the reactors.

### 2.2. Experiments Progress

**Figure 1:**
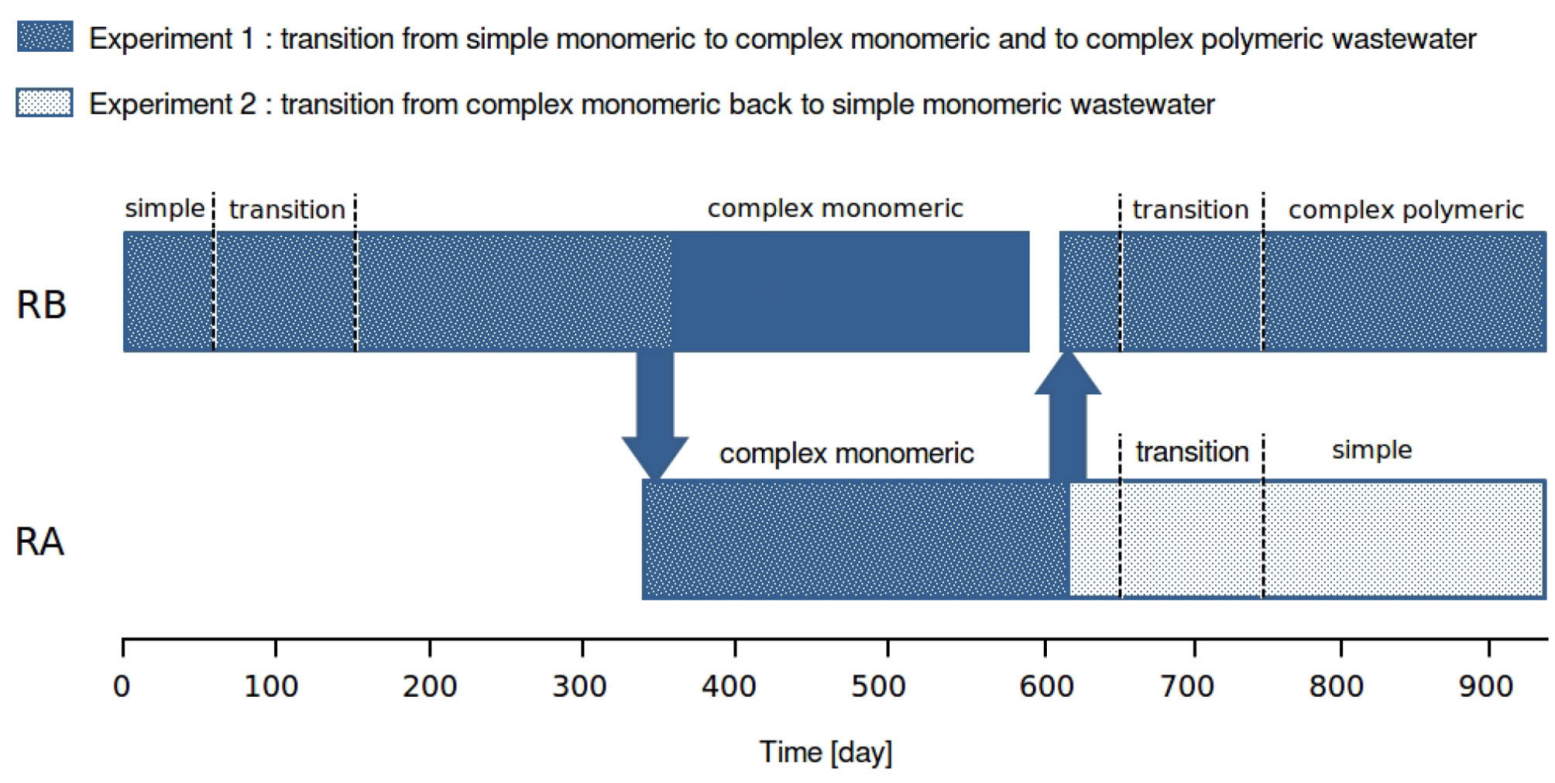
Summarizing diagram of the reactor operation and the two experiments. Experiment 1 started in RB with AGS acclimated to a simple wastewater [containing mainly volatile fatty acids (VFA)]. The wastewater feeding the AGS was progressively replaced by a complex monomeric wastewater (containing VFA, glucose, and amino acids). On day 333, half of the sludge of RB was used to start reactor RA. The two reactors were fed with complex monomeric wastewater. On day 600, RB was stopped and emptied. On day 607, half of the sludge from RA was used to re-start RB. In RB, the complex monomeric wastewater was progressively replaced by a complex polymeric wastewater (containing VFA, glucose, amino acids, starch, and peptone). In RA, the complex monomeric wastewater was progressively replace by a simple wastewater.

Two reactors, RA and RB, were operated (Figure 1). The reactor RB had been initially inoculated in spring 2014 with activated sludge collected in a wastewater treatment plant performing enhanced biological phosphorus removal (ARA Thunersee, Switzerland). The synthetic wastewater used until the beginning of the experiment was composed of acetate and propionate as carbon source in equal proportions for a total COD of 400 mg O_2_ l^−1^, 22 mg l^−1^ of P-PO_4_, 56 mg l^−1^ of N-NH_4_^+^, and trace elements. Experiment 1, designed to investigate separately the effect of fermentable and polymeric compounds on AGS, started in autumn 2015. First, the composition of this simple wastewater was progressively changed to a complex monomeric wastewater by introducing glucose and amino acids. On day 333, the reactor RA was started with half of the sludge from RB and on day 607, the reactor RB was stopped and restarted with half of the sludge from RA. Then the complex monomeric wastewater was progressively changed to a complex polymeric wastewater by replacing part of glucose and amino acids by starch (solubility: 20 mg ml^−1^, Sigma-Aldrich), and a mixture of peptides of different length and amino acids (<10KD, BD Peptone Bacto, Fisher scientific), in equivalent COD proportions. Experiment 2 (Figure 1), designed to assess the reversibility of the changes induced by the first step of experiment 1, started on day 607 in RA. It consisted in progressively changing the composition of the wastewater from complex monomeric back to simple. Further details about the transitions are provided in Supplementary Material (Complement 1).

### 2.3. Wastewater Composition

The influent wastewater was a mixture of 0.135 l of concentrated autoclaved C-medium, 0.135 l of concentrated NP-medium and 0.93 L of water pumped form lake Geneva filtered at 100 μm from day 0 to 515, and at 5 μm from day 515 to day 925. An aliquot of 50 ml trace element solution containing 16.22 g l^−1^ of C_10_H_14_N_2_Na_2_O_8_ · 2H_2_O, 0.44 g l^−1^ of ZnSO_4_ · 7H_2_O, 1.012 g l^−1^ of MnCl_2_ · 6 H_2_O, 7.049 g l^−1^ of (NH_4_)_2_Fe(SO_4_)_2_ · 6 H_2_O, 0.328 g l^−1^ of (NH_4_)_6_Mo_7_O_24_ · 4 H_2_O, 0.314 g l^−1^ of CuSO_4_ · 5H_2_O and 0.322 g l^−1^ of CoCl_2_ · 6H_2_O was added to the NP-medium for a final volume of 10 l. The resulting characteristics of the different influent synthetic wastewaters are presented in Table 1.

**Table 1:** Theoretical characteristics of the different influent synthetic wastewater in mgl^−1^.

**Denomination**	**Medium number**	**COD (mg O_**2**_l^**−1**^)**	**VFA (% of COD)**	**Glucose**	**Starch**	**Amino acids**	**Peptone**	**TKN (Nl^**−1**^)**
Simple	1	456	100	–	–	–	–	56
Transition to	2	448	82	9	–	9	–	63
	3	439	62	19	–	19	–	69
	4A	427	36	32	–	32	–	78
Complex monomeric	4B	600	33	33	–	33	–	89
	4C	600	33	33	–	33	–	56
Transition to	5	600	33	28	5	28	5	56
	6	600	33	23	10	23	10	56
Complex polymeric	7	600	33	17	17	17	17	56
Transition back to	3B	600	60	20	–	20	–	56
	2B	600	80	10	–	10	–	56
simple	1B	600	100	–	–	–	–	56

*Simple wastewater was mainly composed of VFA, complex monomeric wastewater contained VFA, glucose and amino acids and complex polymeric wastewater contained VFA, glucose, amino acids, starch and peptone. The theoretical concentration of phosphate was 22 mgl^−1^ of ${\text{P-PO}}_{4}^{-}$ in all the wastewaters. The composition of the different C- and NP-media are presented in Supplementary Table 1*.

### 2.4. Nutrient-Removal Performance Monitoring

The nutrient removal performance of the reactors was measured on a bi-weekly basis on the influent wastewater (I), the water collected from the reactor at the end of the anaerobic phase (AN) and at the end of the aerobic phase (S). Water samples were filtered at 0.45 μm and stored at 4°C before further analysis. Ammonium concentrations were measured by spectrophotometry, using two different kits; the ammonia cuvette test (0.015–2.0 mg l^−1^ ${\text{N-NH}}_{4}^{+}$, LCK304, Hach, USA) for the samples with an ammonia concentration estimated below 3 mg l^−1^ of ${\text{N-NH}}_{4}^{+}$, and the Spectroquant ammonium test (photometric 0.010–3.00 mg l^−1^ ${\text{N-NH}}_{4}^{+}$, Merck, Germany) for the other samples^1^. The concentrations of anions (${\text{P-PO}}_{4}^{-}$, ${\text{N-NO}}_{3}^{-}$, and ${\text{N-NO}}_{2}^{-}$) were measured by ionic chromatography (IC, ICS-90, IonPacAS14A column) with an electrical conductivity detector (Dionex, Switzerland).

### 2.5. Measures of SVI, TS, and VS

The sludge volume index (SVI), the volatile solids (VS), and the total solids (TS) were measured according to the standard method (APHA, 2012). Approximately 500 ml of mixed liquor were collected from the reactor during mixing. The volume occupied by the sludge bed was measured in a graduated cones and photographs were taken after 3, 5, 8, 10, and 30 min. This mixed liquor was mixed again and three aliquotes of ~20 ml were collected in falcon tubes and centrifuged at 8,392 × g during 5 min. For each aliquote, the mass of the dried pellet after 12 h of drying at 105°C gave the TS and the mass loss after 2 h of calcining at 550°C gave the VS. The SVI corresponding to the different times were calculated as the ratio between the volume of the sludge bed (*V*) and the total volume (*V*_tot_), divided by the mean TS of the triplicates (Equation 1).

(1) $$\text{SVI}(\text{ml }{\text{g}}^{-1})=\frac{V(\text{ml})}{\text{TS}(\text{ml }{\text{g}}^{-1})\cdot V_{\text{tot}}(\text{ml})}$$

### 2.6. Nematodes Monitoring and Controlling

The presence and abundance of nematodes in the sludge was monitored by visual inspection with light microscopy (Eclipse NI-U Microscope, Nikon). In case of too high abundance of nematodes, their number was reduced by centrifuging the biomass with an Avanti J-26 XPJ (Beckmann Coulter) with a JLA rotor (8.1000, Beckmann Coulter) at 15,000 × g and then crushing this biomass with a glass homogenizer having a distance between the pestle and the tube between 0.15 and 0.25 mm (50 cm^3^, Carl Roth, Germany). The homogenized biomass was put back into the reactor and the settling time was adapted to the temporarily decreased settling properties. This procedure, hereafter referred to as “homogenization of the sludge,” was applied on the sludge of the reactor RB on days 560 and 798 and of the reactor RA on day 539.

### 2.7. Biomass Sampling

AGS biomass was collected from the reactor(s) on a weekly basis according to the following procedure. Mixed liquor containing between 1 and 2 ml of wet biomass was centrifuged during 5 min at 8,392 × g (Nuaire Awel CF-48R centrifuge, USA) and washed twice with 5 ml of phosphate buffer saline (PBS, 8 g l^−1^ of NaOH, 0.2 g l^−1^ of KCl, 1.44 g l^−1^ of Na_2_HPO_4_, 0.24 g l^−1^ of KH_2_PO_4_) at 4°C followed by 5 min of centrifugation at 8,392 × g. The pellet was resuspended in 3 ml of PBS, homogenized with a glass homogenizer with a distance between the pestle and the tube between 0.15 and 0.25 mm (5 cm^3^, Carl Roth, Germany) and distributed in five cryotubes. The samples were stored at −20°C until DNA extraction. When a sufficient proportion of the biomass was in the form of flocs, separate granules and flocs samples were collected in supplement of the mixed sample, by using a 250 μm sieve before washing and homogenization.

### 2.8. DNA Extraction and 16S rRNA Gene Sequencing

For each sample, 200 μl of homogenized biomass was mixed with 400 μl of elution buffer (T_10_E_0.1_) and 100 μl of lysozyme solution (25 mg ml^−1^). After 1 h at 37°C, DNA was extracted by using the automatic robot 16 DNA purification system (Maxwell, Promega Corporation, Switzerland). The DNA concentration of each DNA extraction was determined by spectrophotometry with a NanoDrop (ND1000, Witec AG, Switzerland).

For reasons out of our control, two different Illumina MiSeq sequencing platforms, and therefore two slightly different protocols were used for 16S rRNA gene amplicon sequencing. In both cases, the hypervariable regions V1–V2 of the bacterial 16S rRNA gene were amplified by polymerase chain reaction (PCR) using the universal bacterial primers 27F and 338R (see sequence below). The amplified and barcoded DNA was sequenced in multiplexed by groups of 60 (run1) or 96 samples (other runs), in paired-end mode (2x250 bp).

#### 2.8.1. Protocol n° 1

Runs 1, 2, and 3 (corresponding to samples from days 0 to 385) were performed at the Laboratory of Intestinal Immunology (UPHARRIS, EPFL). The indexing of the sample was performed during the PCR by using a 27F primer and 60 (run1) or 96 (runs 2 and 3) different barcoded 338R primers (in bold) with overhang containing Illumina adapters. 5′GAGATCTACACTATGGTAATTCC-**AGMGTTYGATYMTGGCTCAG**3′ and 5′ CAAGCAGAAGACGGCATACGAGAT-barcode-AGTCAGTCAGAA-**GCTGCCTCCCGTAGGAGT**3′, respectively. The DNA was amplified according to the procedure described in Layer et al. (2019). The amplified DNA quantified on a labCHip GX (Perkinelmer), with 1–2 μl of each PCR product and the kit DNA 5k (CLS760675). The samples were then pooled to a final concentration of 4 nM, purified by using Agencourt AMPure XP beads (Beckman Coulter, A63880) and sequenced.

#### 2.8.2. Protocol n° 2

Runs 7, 8, and 11 (corresponding to samples from days 392 to 925 and some replicates of samples corresponding to day 0–385) were sequenced at the Lausanne Genomic Technologies Facility (GTF), University of Lausanne, Switzerland (https://www.unil.ch/gtf/en/home.html). A first PCR was performed with the 27F and 338R primers (in bold) with overhang adapter sequences 5′TCGTCGGCAGCGTCAGATGTGTATAAGAGACAG-**AGMGTTYGATYMTGGCTCAG**3′ and 5′GTCTCGTGGGCTCGGAGATGTGTATAAGAGACAG-**GCTGCCTCCCGTAGGAGT**3′, respectively. The same procedure as in the protocol n°1 was followed. The PCR products were purified by using Agencourt AMPure XP beads (Beckman Coulter, A63880), quantified with a fragment analyzer using the DNF-473 standard sensitivity NGS fragment analysis kit (Advanced analytical Technologies Inc.). These amplified samples were transmitted to the GTF for a secondary indexing PCR and multiplex sequencing. The raw sequences have been deposited in ENA under the study accession number ERP122292.

### 2.9. Taxonomic Affiliation of 16S rRNA Gene Sequences

The taxonomic affiliation of the 16S rRNA gene sequences was performed similarly to the procedure described (Layer et al., 2019). After demultiplexing, the sequences were trimmed and filtered by using trimmomatic version (v.) 0.36 (Bolger et al., 2014) with a sliding window of 4 base pairs (bp), a quality score threshold of 15 and a minimal length of 100 bp. The paired-end reads were merged with Pear v. 0.9.11 (Zhang et al., 2014).

The sequences were clustered with a minimum similarity threshold of 99% by using Swarm v. 2.2.2 (Mahé et al., 2014) with the commands line options −*t 4* −*d 3* −*z* −*w*. Clusters with at least 5 sequences per sample were kept and their representative sequences were compared with the 16S rRNA gene database MiDAS v. S123_2.1.3 (McIlroy et al., 2017) by using the blast software (Altschul et al., 1990).

The taxonomy of the best match was attributed to all the sequences of the cluster. The level of precision of the taxonomy was adjusted according to the percentage of similarity with the threshold sequence identity values given by Yarza et al. (2014), 94.5% for genus, 86.5% for family, 82.0% for order, 78.5% for class, and 75.0% for phylum.

### 2.10. Statistical Analysis

The statistical analysis and related plots were performed as described (Layer et al., 2019) with R program v. 3.5.0 (R Development Core Team, 2008) using the packages reshape2 and ggplot2 (Wickham, 2007, 2016; Warnes et al., 2016). Correlation data were computed with the function *rcorr* from the package Hmisc v. 4.4-1 (Harrell et al., 2020). Bray-Curtis distance matrices and the associated principal coordinates analysis (PCoA) were produced with the package vegan v. 2.5-2 (Oksanen et al., 2018).

### 2.11. Determination of Stable States and Discriminant Taxa

The Bray-Curtis distance matrix of the bacterial OTU (99%) relative abundance (Supplementary Data Sheet 2) was used to define the bacterial “stable states” of these experiments. A stable state was defined as the maximal cluster of successive samples having a pairwise distance lower than 0.5 (the maximum pairwise distance between two samples was 0.9). After Hellinger transformation, the mean relative abundance of each taxa (at the genus level) was compared, using *t*-tests, between the stable states grouped by influent wastewater types (simple monomeric, complex monomeric, and complex polymeric). The taxa were considered “divergent” if their mean was significantly different (*p*-value 0.01 with Bonferroni correction for multiple testing, *p*-value = 0.01/269 = 3.72 × 10^−5^) between at least two influent wastewater types. These taxa were considered “abundant” if their average abundance was higher than 1% in at least one wastewater type at stable state. The taxa being divergent and abundant were considered as “discriminant” in the following analysis.

### 2.12. Comparison of the Bacterial Communities in Flocs and Granules

The average proportions of the abundant taxa were compared by using *t*-tests within the stable state for which granules and flocs were collected separately for at least four samples. The difference was considered significant if the *p*-value was lower than the Bonferroni corrected *p*-value of 0.01 (*p*-value = 0.01/29 = 3.45 × 10^−4^).

## 3. Results

### 3.1. Nutrient-Removal Performance

**Figure 2:**
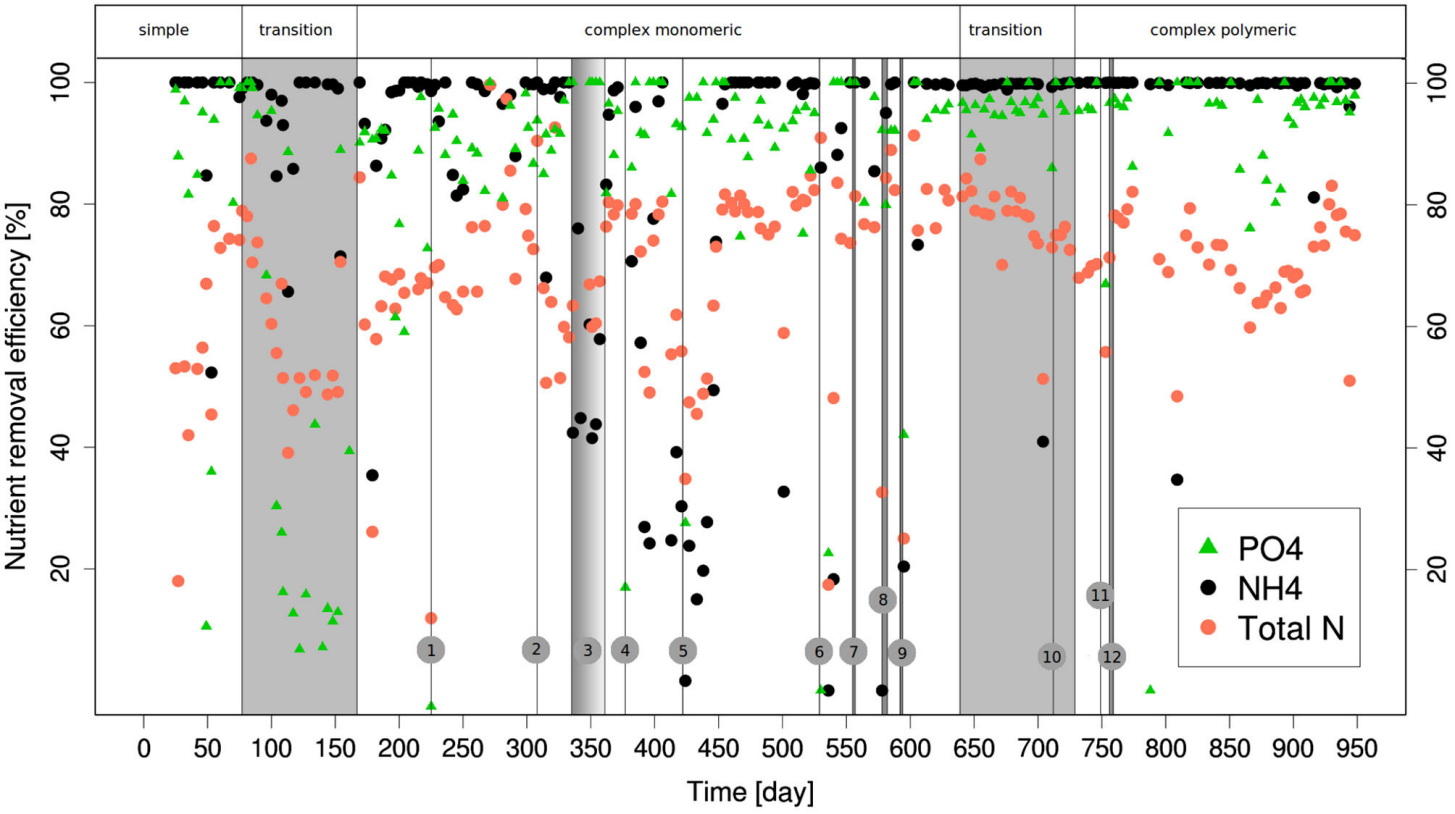
${\text{PO}}_{4}^{-}$-, NH_4_-, and TN-removal performance of the AGS during experiment 1. The process events susceptible to having had a negative impact on nutrient removal performance are numbered from 1 to 12. (1) No C-medium in the influent wastewater during eight cycles. (2) The leak of O_2_ detected since several days is corrected. (3) Few biomass in the reactors due to the duplication of the reactors and the feeding was not adapted. (4) Problem with pumping of NP-medium. (5) The pH of the bulk water was higher than 11 during at least 2 h. (6) The pH of the bulk water was higher than 11 during a period of time estimated to 24 h. (7) Problems with the recirculation pump possibly leading to lower shear stress during 2 days. (8) No trace elements in the influent during 4 days. (9) The influent was composed of twice the NP-medium and 200 mg O_2_ *ml*^−1^ of glucose during 2 days. (10) No feeding during the night. (11) Loss of biomass due to accidental change from 5 to 3 min of settling. (12) Lower concentrations of C-medium in the influent due to malfunctioning of the pumping.

**Figure 3:**
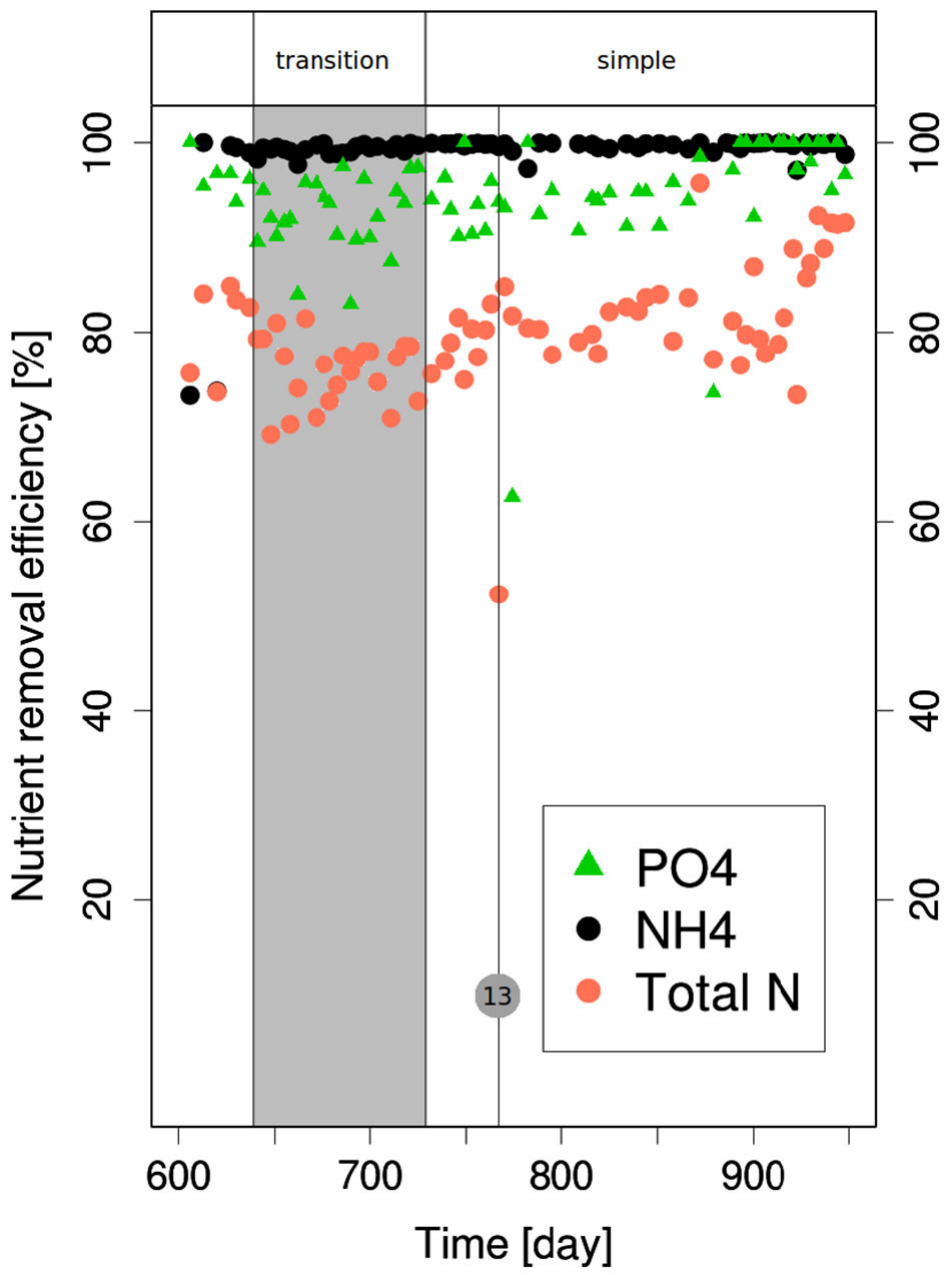
${\text{PO}}_{4}^{-}$-, NH_4_-, and TN-removal performances during the transition back from complex monomeric to simple monomeric influent wastewater. The vertical bar with the number (13) corresponds to the day with no feeding during several cycles.

The overall nutrient removal performances (Figures 2, 3), summarized in Table 2, were quite well-maintained despite the changes of wastewater types. Several operational incidents had major, but only temporary impact on these performances.

**Table 2:** Summary of the AGS average performance in percentage, according to the synthetic wastewater type*^a^*.

**Wastewater type**	**${\text{NH}}_{4}^{+}$-removal**	**N-removal**	**P-removal**
Simple	98.4 ± 7.1	74.9 ± 13.7	90.8 ± 16.0
Complex monomeric	84.6 ± 24.7	70.9 ± 12.6	89.2 ± 16.5
Complex polymeric	97.6 ± 11.2	69.9 ± 6.4	91.7 ± 16.9

a *The performance data corresponding to operation incidents have been removed. These data corresponds to days 225 and 749 in RB and days 377, 424, 427, 530, 536, 578, 581, 595 in RA*.

The NH_4_-removal was complete during most of the experiments. However, temporary decreases of this efficiency were noticed and this happened almost always due to the operational difficulties listed in the legend of Figures 2, 3. Most of these difficulties occurred when the reactors were fed with complex monomeric wastewater. Although the measurements clearly identified as corresponding to an operation incident have not been taken into account for the calculation of the mean, the NH_4_-removal generally took some time get back to its optimal efficiency. N-removal was enhanced after the introduction of the alternated aeration during the aerobic phase at day 48.

It decreased during the first transition due to the supplementary nitrogen input coming from amino acids degradation. With the complex monomeric wastewater, N-removal was on average a bit lower than with the simple wastewater due to the lower NH_4_-removal. The transition from complex monomeric to polymeric wastewater went with a slight decrease of N-removal but this time due to a lower denitrification. P-removal performances decreased to <20% during the transition from simple to complex monomeric wastewater. The increase of the total COD of the wastewater from 427 to 600 on day 145 was followed by a recovery of the P-removal efficiency (higher than 80%). Aside from the operational difficulties mentioned above, the incidents impacting P-removal were linked to the presence of O_2_ in the anaerobic phase (on day 308) or to incidents causing a lower COD concentration in the wastewater (on day 313 and 751).

### 3.2. Settling Properties

The SVI_3_ measures the volume occupied by 1 g of sludge after 3 min of settling and the ratio SVI_30_/SVI_10_ indicates how much the biomass changed of volume between 10 and 30 min of settling. They were measured on the AGS during the transition from simple to complex monomeric wastewater and to complex polymeric wastewater (Table 3) in order to assess the evolution of its settling properties during these two changes of wastewater composition. At the beginning of the experiment, the AGS was composed almost exclusively of granules and had excellent settling properties with a sludge volume index after 3 min (SVI_3_) below 25 ml g^−1^ and a SVI ratio of 1. The introduction of amino acids and glucose in the wastewater did not have a big impact on the settling properties. Indeed, the SVI_3_ was still below 25 ml g^−1^ and the SVI ratio was still equal to 1, on day 124. However, the proportion of large granules became slightly lower between day 384 and 482 and the AGS did not recover its initial size distribution during these two experiments (Supplementary Figure 1).

**Table 3:** Settling characteristics of the sludge during the different transition phases.

**Day**	**Wastewater type**	**SVI_3_**	**SVI_30_**	**SVI_3_/SVI_10_**
**TRANSITION FROM SIMPLE TO COMPLEX MONOMERIC**				
57	Simple	18.5	–^a^	1
95	Simple	18.5	–^a^	1
109	Transition	18.7	–^a^	1
124	Transition	22.4	22.4	1
614	Complex monomeric	35.5	30.5	0.93
**TRANSITION FROM COMPLEX MONOMERIC TO COMPLEX POLYMERIC**				
680	Complex monomeric	35.5	34.5	0.97
729	Transition	100.2	61.2	0.81
770	Complex polymeric	149.1	71.5	0.75
886	Complex polymeric	189.0	92.0	0.71
910	Complex polymeric	126.0	66.6	0.76
**TRANSITION FROM COMPLEX TO SIMPLE MONOMERIC**				
680	Complex monomeric	44.6	40.7	0.99
729	Transition	42.0	39.5	0.98
770	Simple	49.1	47.9	1
886	Simple	68.0	60	0.98
910	Simple	55.8	51	0.97

a *The sludge volume index after 30 min (SVI_30_) were only measured from day 124 on*.

The introduction of polymeric compounds was followed by a big increase of the sludge volume index. At first, the proportion of granules progressively decreased almost to zero and the biomass became mostly floccular (Supplementary Figures 2C,D). In the following, a sudden loss of 30% of volume without loss of biomass and the visual observation of numerous small granules on day 788 indicated a granulation restart, but a rather high proportion of flocs (around 20%) remained until the end of the experiment (Supplementary Figure 2E). Even then, the SVI_30_ remained below 100 ml g^−1^.

During the transition from complex back to simple monomeric wastewater, the settling properties of the AGS decreased slightly but the SVI_3_ remained around 60 ml g^−1^ and the SVI ratio was above 0.9.

### 3.3. Stable States

**Figure 4:**
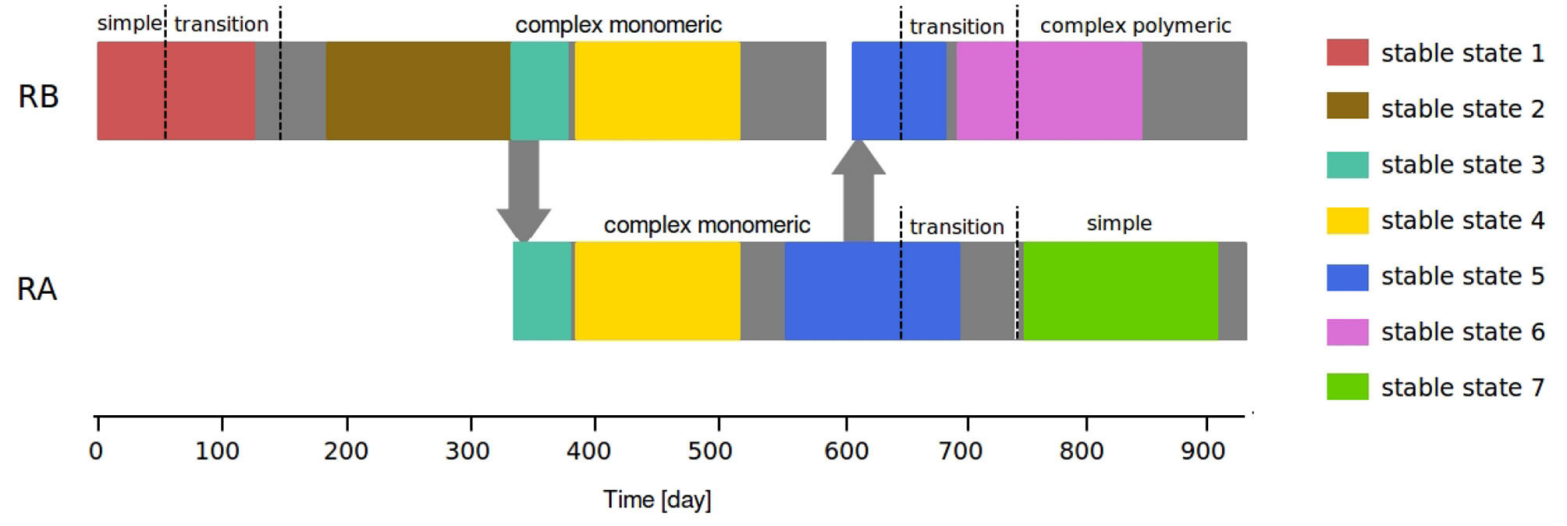
The seven bacterial stable states detected during experiment 1 and 2.

The changes of wastewater composition, along with other factors induced changes in the bacterial community composition of the AGS, generally observed with a lag of 1–2 weeks. Seven different “stable states” were detected in the bacterial communities in the AGS of experiment 1 and 2 (Figure 4). The average bacterial community composition of each stable state is presented in Supplementary Figure 3. The first stable state was observed with the simple monomeric wastewater and persisted during 6 weeks of transition. The second stable state begins 3 weeks after that the transition to monomeric wastewater was complete and persisted until the inoculation of the second reactor (RA). This second state was characterized by a particularly low proportion of *Ca*. Accumulibacter and a high proportion of *Ca*. Competibacter (Supplementary Data Sheet 3). The third stable state also corresponds to complex monomeric wastewater feeding and was detected in the two reactors in parallel. It began after the splitting of the biomass in two reactors and lasted 6 weeks, until the usual amount of biomass was recovered.

Shortly afterwards, a forth stable state was observed in parallel in the two reactors. The end of this stable state correspond to a period of time when a lot of biomass was sampled from the reactors for different experiments, followed by operation failures, such as a pH regulation incidents (RA) or the lake of trace elements in the influent (RA and mainly RB). The fifth stable state started in RA on day 558, several weeks after the stabilization of the reactor operation. It persisted in RB after inoculation and during the first 7 weeks of the transition to polymeric wastewater and in RA during the first 8 weeks of transition to simple wastewater. The sixth stable state was observed during the second part of the transition to complex polymeric wastewater and persisted during 4 months and a half. The seventh began 1 week after that the transition from complex back to simple monomeric wastewater was complete and persisted during 5 months.

**Figure 5:**
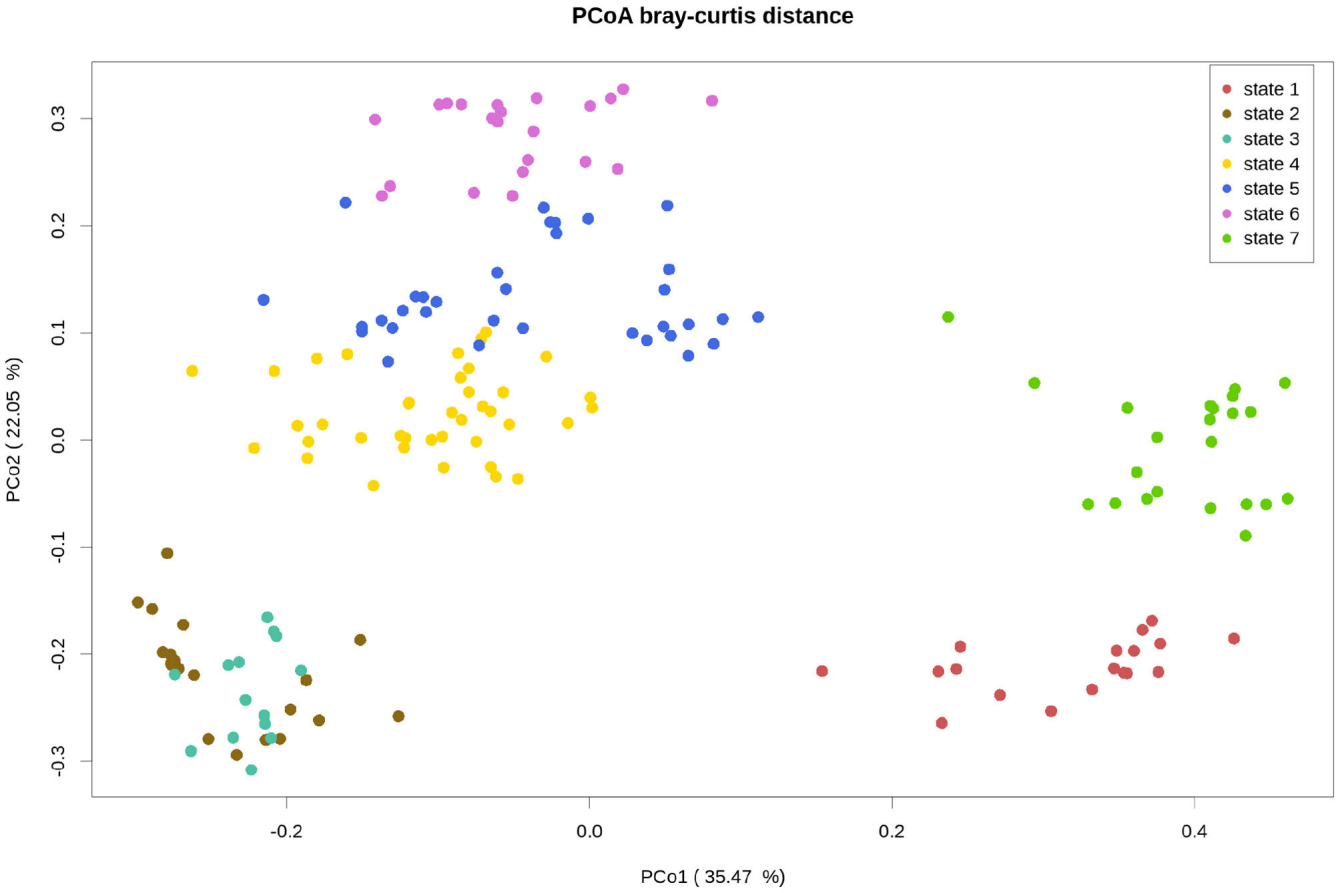
Principal component analysis based on the Bray-Curtis distance of the bacterial communities (genus level) at stable states. The two first coordinates PCo1 and PCo2 explain 35.47 and 22.05% of the variance, respectively. The plots of the three first coordinates are provided in Supplementary Figure 4.

The main similarities between the bacterial communities are highlighted in the PCoA based on their Bray-Curtis distance matrix of the samples at stable state (Figure 5). On the two first axis of this PCoA, the stable states are grouped in tree big clusters : stable states 1 and 7 (simple monomeric), stable states 2 and 3 (complex monomeric), and stable states 4, 5 (complex monomeric) and 6 (complex polymeric).

The bacterial communities from stable states 1 and 7, corresponding to the simple wastewater before and after the passage to complex monomeric wastewater, are close to each other, but they are not overlaid. This confirms that the changes in the bacterial communities induced by this change of wastewater composition were only partly reverted. This can also imply that the bacterial communities respond to factors other than the composition of the wastewater alone. Stable states 2 and 3 are clearly separated from the rest of the samples on the second axis, although they were fed with the same type of wastewater as the samples from stable states 4 and 5. The difference between the bacterial communities of these two stable states may be primarily due to the low abundance of *Ca*. Accumulibacter characterizing these bacterial communities. The stable states 4, 5, and 6 are closely related in this analysis. The differences between the samples collected before and after the transition from complex monomeric to complex polymeric wastewater do not show on the first axis of this PCoA, revealing that the changes of bacterial communities during this transition were small compared to the changes induced by other wastewater composition changes or perturbations.

### 3.4. Changes in the Microbial Communities During the Transition to a Complex Polymeric and Back to Simple Wastewater

**Figure 6:**
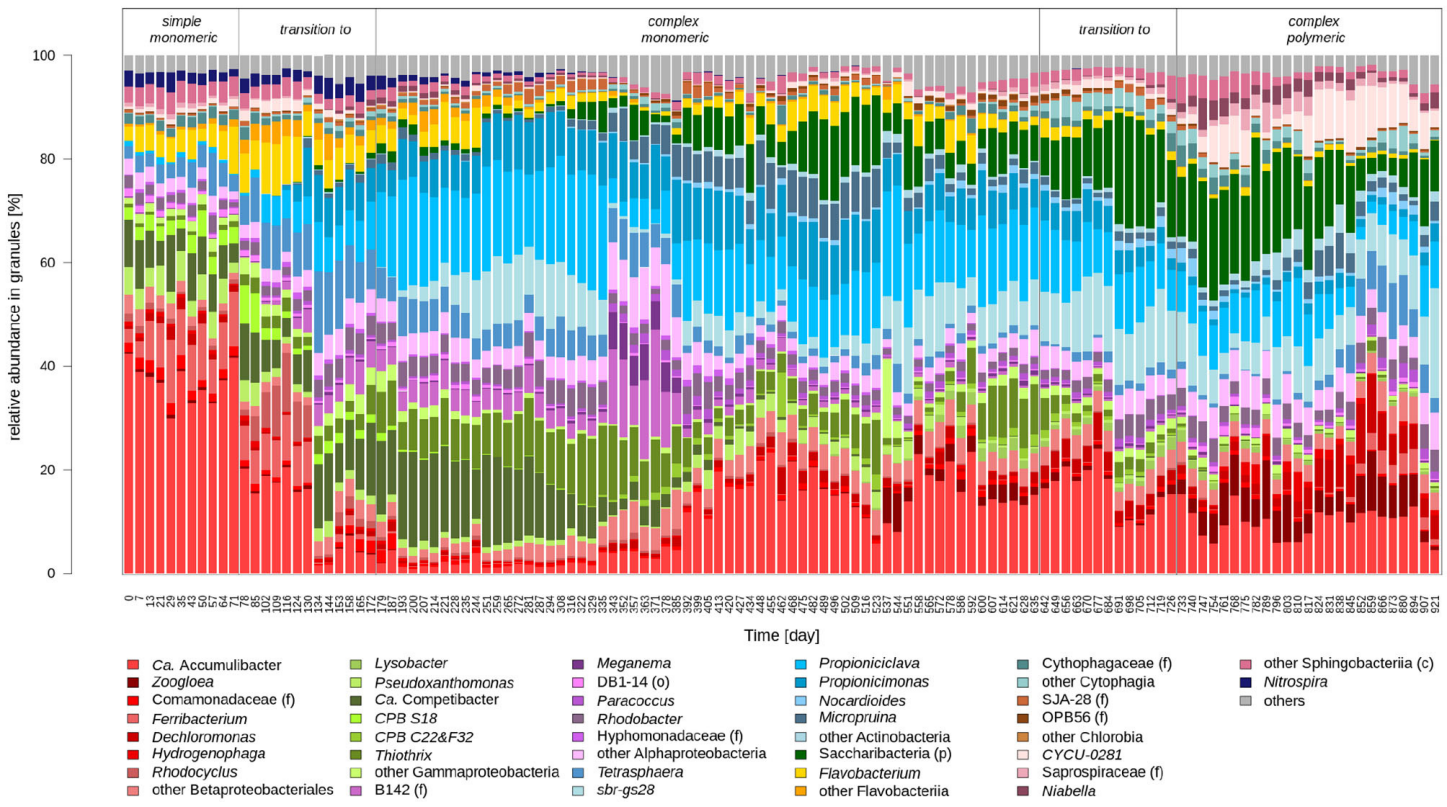
Evolution of the bacterial community composition during the transition from simple to complex monomeric wastewater and to complex polymeric wastewater. The genera are colored according to the class they belong to, with the exception of the Betaproteobacteriales, previously the class of Betaproteobacteria, colored in red. They were recently merged with the Gammaproteobacteria (Parks et al., 2018), here colored in light green. Only the abundant bacteria (mean > 1% with at least one wastewater type) are shown.

**Figure 7:**
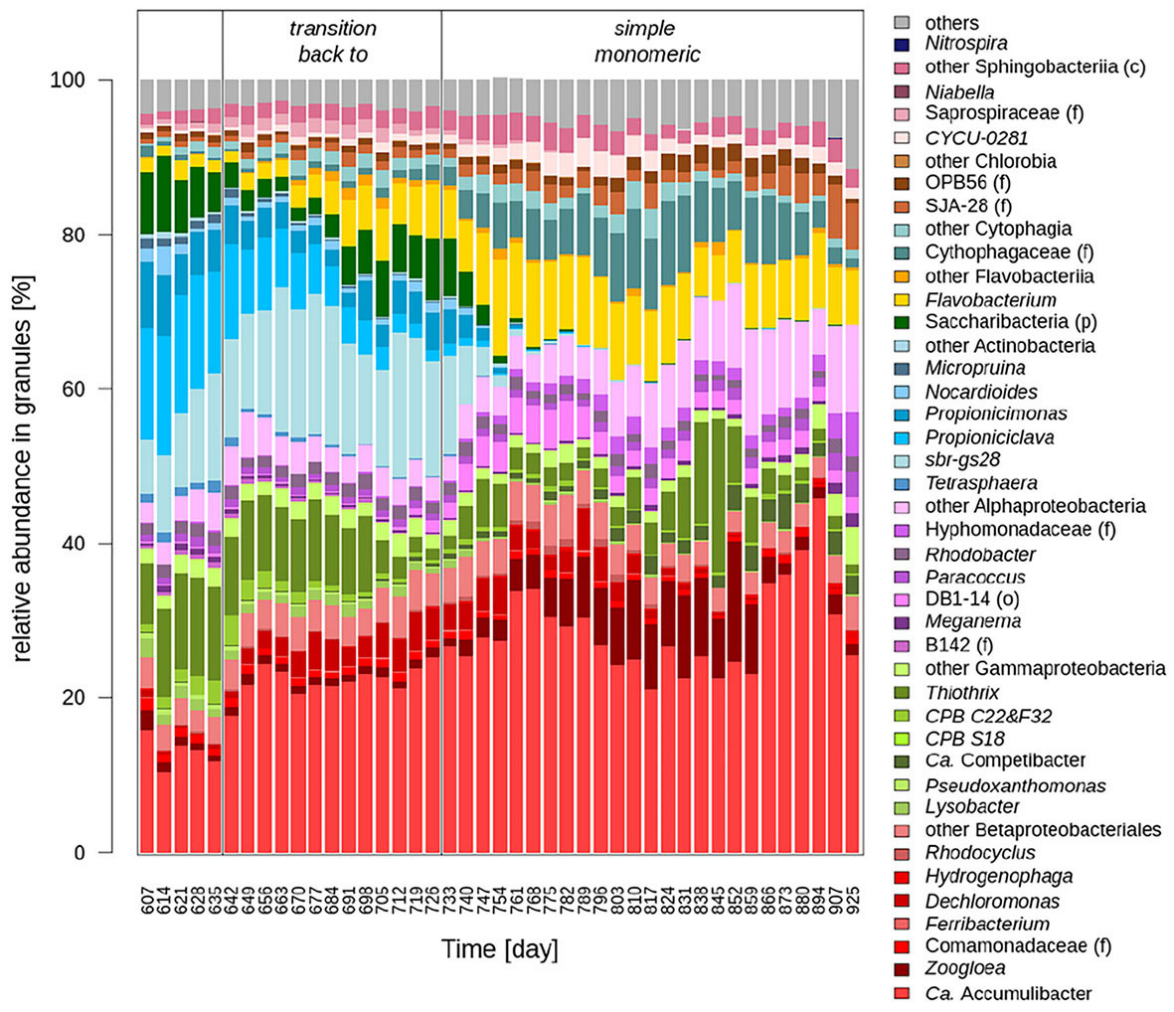
Evolution of the bacterial communities during the transition back from complex monomeric to simple wastewater. The genus are colored according to the class they belong to, with the exception of the Betaproteobacteriales, colored in red, whereas the other Gammaproteobacteria are colored in light green. Only the abundant bacteria (mean > 1% with at least one wastewater type) are shown.

The time series of the bacterial community composition, followed during 921 days of experiment 1 and 318 days of experiment 2 by 16S rRNA gene amplicon sequencing are presented in Figures 6, 7, respectively. During the first transition from simple to complex monomeric wastewater, the relative abundance of Gammaproteobacteria decreased to the benefit of Actinobacteria. In a first time, *Ca*. Accumulibacter (*p*-value 4.2 × 10^−13^, Supplementary Data Sheet 3), the bacterial genus dominant in AGS fed with the simple wastewater, decreased abruptly and the proportion of *Ca*. Competibacter (*p*-value 2.0 × 10^−5^) increased. The genus *Nitrospira* was present at around 3% with the simple monomeric wastewater, and it progressively decreased below 1% (*p*-value 1.6 × 10^−10^) after the transition to complex monomeric wastewater. During the weeks following the start of the second reactor RA with half of the sludge of the reactor RB, on day 333, the amount of biomass per reactor was decreased by a factor of two and the food to microorganism ratio was higher during this period. The relative abundance of bacteria from the class of Alphaproteobacteria increased from 20 to 40% for 7 weeks. Afterwards, the relative abundance of *Ca*. Accumulibacter increased again (*p*-value 2.1 × 10^−1^) and remained around 20%, and those of *Tetrasphaera* (*p*-value 1.5 × 10^−13^) and *Ca*. Competibacter (*p*-value 2.8 × 10^−8^) decreased below 4%. Uncharacterized bacteria from the phylum of Saccharibacteria appeared in the system during the transition to complex monomeric wastewater and progressively reached a relative abundance around 10%. After the second pH incident, on day 529, the relative abundance of Zoogloea increased (*p*-value 2.5 × 10^−14^) up to 10% and remained around 5% during the following weeks.

The transition from complex monomeric to polymeric wastewater had a smaller effect on the bacterial community composition than the transition form simple to complex monomeric wastewater. The relative abundance of Saccharibacteria was around 20% during the transition and the three following months. In the class of Gammaproteobacteria, the proportion of *Ca*. Accumulibacter decreased (*p*-value 1.1 × 10^−9^) and stabilized around 10%, whereas the proportion of *Zoogloea* increased (*p*-value 1.2 × 10^−4^). On day 848, corresponding to the formation of new granules, the proportions of *Dechloromonas, Zoogloea*, and *Tetrasphaera* were around 10% each. The proportion of *Thiothrix*, which represented around 7% of the biomass with the complex monomeric wastewater, decreased to <1% with the complex polymeric wastewater (*p*-value 5.1 × 10^−13^), whereas the one of the class of Sphingobacteria increased up to around 10%.

The reversibility of the changes of the bacterial community induced by the transition from simple to complex monomeric wastewater was assessed by performing the reverse transition in reactor RA. After this transition from complex monomeric back to simple monomeric wastewater, the bacterial community was similar to the one composing the AGS at the beginning of experiment 1, but several differences were observed. Although Gammaproteobacteria had a similar abundance (60%) as at the beginning of experiment 1 (70%), *Zoogloea* (*p*-value 2.9 × 10^−10^) and *Thiothrix* (*p*-value 2.7 × 10^−5^) were more abundant after the transition back to simple monomeric wastewater whereas *Ca*. Competibacter (*p*-value 4.7 × 10^−11^) *Ferribacterium* (*p*-value 2.6 × 10^−14^) were less abundant. The Actinobacteria, whose proportion had increased during the change to complex wastewater, became even less abundant (<1%) after the change back to simple monomeric wastewater than they were initially. Also the classes of Alphaproteobacteria, Chlorobia, and Cytophagia were more abundant than they were at the beginning. After day 894, new changes were noticed in the bacterial community composition, with in particular, the decrease of the relative abundance of Zoogloea.

### 3.5. Discriminant Taxa

In order to determine which taxa were the most impacted by the changes of substrate, *t*-test analysis were performed comparing the Hellinger transformed mean relative abundance of the bacterial communities in the stable states of the three influent wastewater types (Supplementary Table 2). There were 34 abundant and 200 divergent taxa (at genus level) on a total of 269. Among the 34 abundant taxa, *Paracoccus, Ferribacterium, Meganema*, and uncharacterized taxa from the families of Comamonadaceae and SJA-28 were not divergent. Thus, they were not significantly impacted by the changes of wastewater composition. *Ca*. Competibacter, *Rhodocyclus*, and *Flavobacterium* were enriched in the monomeric wastewaters. *Ca*. Accumulibacter, *Flavobacterium*, and Bacteria from the families OPB56, Hyphomonadaceae and Cytophagaceae were significantly less present in the AGS fed with the complex wastewater than in the AGS fed with the simple wastewater (Supplementary Table 2). The Actinobacteria *Nocardioides, Propioniciclava, Micropruina*, and *Propionicimonas*, able to grow by fermentation, were significantly enriched in the complex wastewaters. This was also the case for *Rhodobacter, Niabella*, and Saccharibacteria (phylum). The proportion of *Niabella*, Saprospiraceae (family), *Lysobacter*, and Saccharibacteria (phylum) was significantly higher after the introduction of polymers in the wastewater. On the contrary *Thiothrix, Rhodocyclus*, and *Flavobacterium* were significantly less abundant with the introduction of polymeric compounds in the wastewater.

### 3.6. Functional Redundancy

The functional redundancy of the AGS identified bacterial genera, according to MiDAS database (McIlroy et al., 2020), in the different stable states, is shown for the guilds of fermenting bacteria and PAO in Supplementary Figures 5, 6, respectively.

The total proportion of fermenting genera varies between 17.3% (stable state 7, simple wastewater) and 42.1% (stable state 2, complex monomeric wastewater). Among this guild, the proportion of Actinobacteria was higher in the complex stable states. The taxa that were not identified at the genus level are not shown in this figure. It is, for example, the case for unidentified bacteria of the Saccharibacteria phylum.

The total proportion of PAO varies between 10.2% (stable state 2, complex monomeric wastewater) and 36.4% (stable state 1, simple wastewater). *Ca*. Accumulibacter represents more than 60% of the PAO in all the stable states but stable states 2 and 3. In those stable states *Tetrasphaera* represents 30–40% of the PAO, whereas it is <20% in stable states 1, 4, 5, and 6 and <1% in stable state 7.

### 3.7. Correlation Between the Bacterial Communities in Granules and Flocs

**Figure 8:**
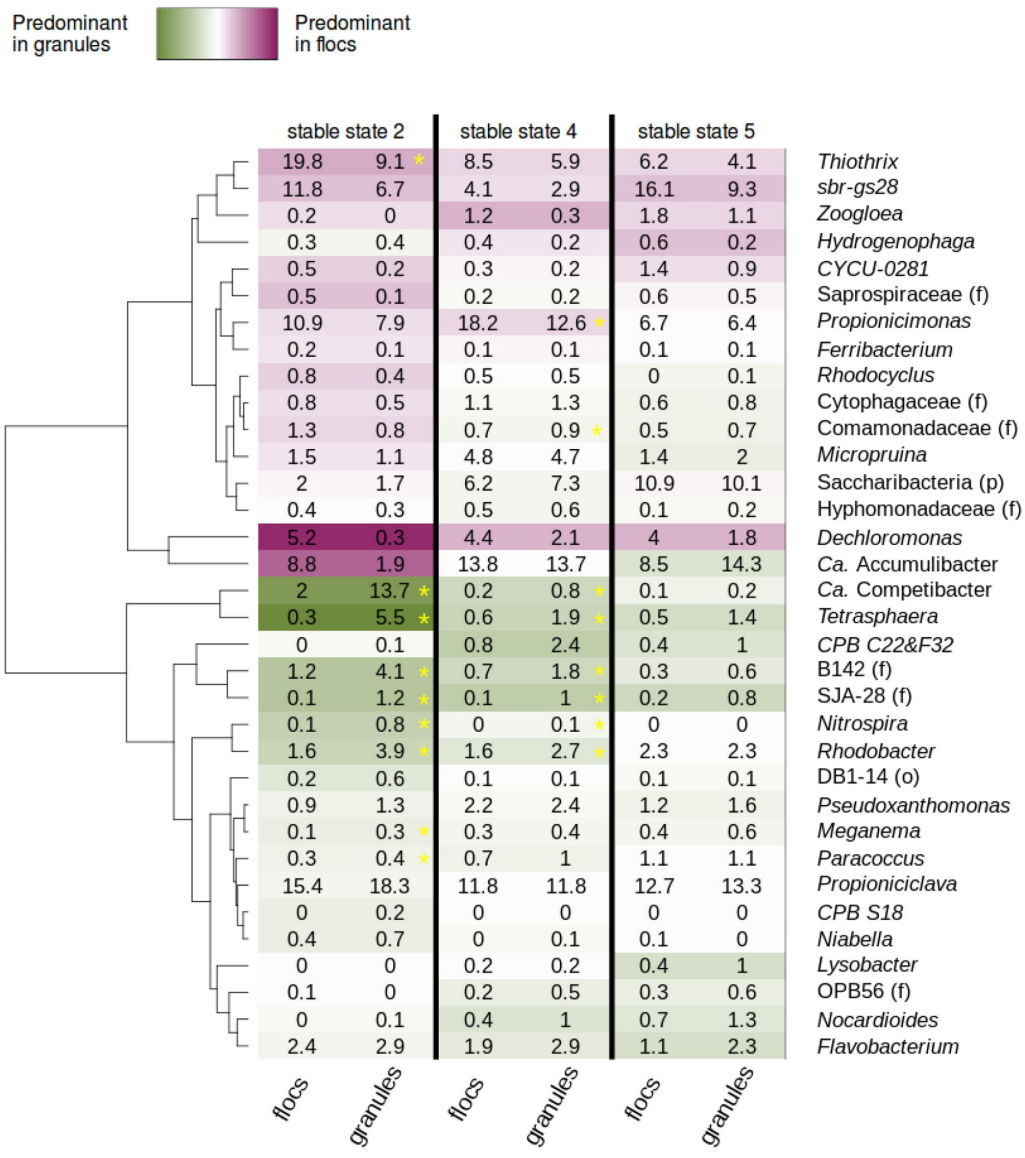
Granules and flocs heatmap showing the average relative abundance of the abundant taxa in AGS fed with complex monomeric wastewater during stable states 2, 4, and 5. The flocs and granules data of stable state 3 were too sparse for this analysis. Green indicates a higher proportion in granules, purple a higher proportion in flocs. In order to lower the possible effect of noise in the very low abundant taxa, a pseudo-count of 0.5% was added to each abundance for the color code and the significance analysis. A yellow asterisk indicates the differences that are significant with an adjusted *p*-value of 2.94 × 10^−4^ (0.01/34).

The proportions of the abundant taxa in the flocs and granules were compared in order to determine potential enrichment in one fraction or the other (Figure 8). Stable states 2 and 4 gather, respectively 14 and 21 observations, whereas stable state 5 gather nine observations. The low amount of data can be the cause for the absence of significant results for this latter state. For the majority of the taxa, no strong tendencies are detected. In the stable states 2, 4, and 5, *Tetrasphaera*, the GAO *Ca*. Competibacter, *CPB_C22*&*F32*, SJA-28 (f), *Nocardioides, Flavobacterium*, and B142 (f) were enriched in granules, whereas *Thiothrix*, the phylotype sbr-gs28, *CYCU-0281, Zoogloea, Propionicimonas*, and *Dechloromonas* were enriched in flocs.

### 3.8. Correlation Between the Bacterial Communities and the Nutrient Removal Performances

Correlations (Pearson) between the nutrient removal performance and the discriminant taxa are shown in Supplementary Figure 7A, Supplementary Tables 3, 4. Few of these correlations are strong and significant. Yet the main reasons for nutrient-removal performance decreases were identified as being mostly operational dysfunctions. The variable showing the most significant correlations with the relative abundance of the discriminant taxa is the level of phosphate in the bulk water at the end of the anaerobic phase.

The classical PAO *Ca*. Accumulibacter and the classical GAO *Ca*. Competibacter and *CPB_S18* are correlated with high PO_4_ concentrations at the end of the anaerobic phase, whereas the Actinobacteria *Norcardioides, Propionicimonas, Propionicicalva*, the fermenting GAO *Micropruina* and the fermenting PAO *Tetrasphaera* and uncharacterized bacteria of the phylum Saccharibacteria are correlated with low PO_4_ concentrations at the end of the anaerobic phase. With the exception of sbr-gs28 and the Saprospiraceae family, the bacteria correlated with high PO_4_ concentrations were present in higher abundance in the AGS fed with simple wastewater than in the AGS fed with complex wastewater (Supplementary Figure 7B). In the other way around, the bacteria correlating with low PO_4_ concentrations were present in higher abundance in the AGS fed with complex wastewater than in the AGS fed with simple wastewater.

The PAO *Ca*. Accumulibacter as well as the GAO *CPB_C22*&*F32*, sbr-gs28, and *Micropruina* are correlated with high P-removal while the PAO Tetrasphaera and the GAO *Ca*. Competibacter and *CPB_S18* are correlated with low P-removal. Indeed, the relative abundance of these later bacteria was above 5% during the transition from simple to complex monomeric wastewater when *Ca*. Accumulibacter proportion had suddenly decreased. At that time, the P-removal performances had been impacted for several weeks.

Correlations between the different genera and N-removal was generally weak. Yet, most of the bacteria found in higher proportion in the AGS fed with monomeric wastewater are correlated with high N-removal. This correlation can therefore be linked with the wastewater type. Indeed, N-removal was slightly lower with the complex polymeric than with the monomeric wastewater.

## 4. Discussion

### 4.1. Multiple Stable States and Functional Redundancy

Seven stable states were observed on the three types of wastewater. During 15 months in RB and 10 months in RA of reactor operation with complex monomeric wastewater, four different stable states were observed. Perturbations such as change in food over microorganism ratio (stable state 3), too high pH and lack of trace elements (stable state 5), were detected as potential trigger factors for the switch to new equilibria in the bacterial community. Multistability is observed when a small perturbation can lead to a different equilibrium state that will persist even when the external conditions are back to normal (Petraitis and Dudgeon, 2004). The observed transient stable states are possibly a cause of multistability, but it is difficult to ascertain since it is not easy to guarantee that the operating conditions were exactly the same before and after the perturbation (Faust et al., 2015; Gonze et al., 2017). In case of multistability, the non-reversibility of a stable state after a perturbation can be due to stochastic effects, or other factors, such as the priority effect, which describes the exclusion of new arrivals by the bacteria already present in a specific niche (Andersson et al., 2014). Yet the fourth stable state was observed in the two reactors running in parallel from 50 days. This observation excludes purely stochastic drift as the main factor of changes in these microbial communities. The four stable states observed in the reactors treating complex monomeric wastewater were performing similarly in terms of settling and nutrient removal efficiency. Some differences were noted regarding the amount of P-release during the anaerobic phase, testifying of slightly different global metabolism. Most losses in nutrient removal capacity corresponded to operation failures, and no strong correlation could be establish between particular taxa and nutrient removal performance (> −0.42; <0.4). Similarly, microbial communities from conventional or EBPR activated sludge or methanogenic bioreactors were also reported as extremely dynamic in terms of composition without that the performance were affected (Fernandez et al., 2000; Wang et al., 2010; Wells et al., 2011). The functional stability noticed in the present study was likely possible through a sufficient functional redundancy present in the bacterial community before each perturbation (Botton et al., 2006). At the beginning of the experiment, *Ca*. Accumulibacter was the dominant PAO, but *Tetrasphaera* was also present in the system. Therefore, after the sudden decrease of *Ca*. Accumulibacter, *Tetrasphaera* probably took over and P-removal was maintained. The co-existence of these two PAO occupying different niches was previously reported as an important factor of P-removal stability in the EBPR process (Nguyen et al., 2011).

### 4.2. Reversibility of the Changes Induced in the Bacterial Communities

The changes in the bacterial community noticed after the transition from simple to complex monomeric wastewater were only partly reversed after the transition back to simple wastewater. The total proportion of bacteria from the class of Actinobacteria and the phylum of Saccharibacteria, that likely have a fermentative metabolism and benefited from the introduction of glucose and amino-acids in the influent, decreased again below 0.5%. It was expected that fermentative bacteria decrease drastically if the fermentable compounds are not provided anymore. Yet, *Tetrasphaera* was among the abundant bacteria during the initial phase with simple monomeric wastewater. Members of this genus have the capability to take up acetate under anaerobic conditions (without P-cycling) (Nguyen et al., 2011; Kristiansen et al., 2013), allowing them to maintain themselves in AGS fed with simple wastewater. This did not allow *Tetrasphaera* to stay abundant in the AGS after the transition back to simple monomeric wastewater. The difference in the bacterial communities of AGS fed with the simple wastewater before and after the changes of wastewater composition can be explained by the existence of multiple equilibria or also by the difference of the total COD concentration between the two simple wastewaters.

The increase of food to microorganism (F/M) ratio from day 333 was likely beneficial to Alphaproteobacteria and detrimental to Actinobacteria (sbr-gs28 and *Propioniciclava*). But the equilibrium between these two classes was restored when the F/M ratio went back to its previous value. However, inside the class of Actinobacteria, *Micropruina* seemed to benefit from this perturbation to the detriment of the phylotype sbr-gs28 (McIlroy et al., 2020), even when the initial conditions where restored. Similarly, the pH incident on day 529 had a negative effect on several bacterial taxa (*Thiothrix*, Competibacteraceae, Saccharibacteria, *Micropruina, Propionicimonas*). Other taxa, such as *Zoogloea, Propioniciclava*, or Flavobacteriia where less affected and their proportion increased following this event. The proportion of *Zoogloea* diminished during the next week but remained higher than before the perturbation. Therefore, the impact of the pH incident on the bacterial community was only partly reversible.

### 4.3. Transition From Simple to Complex Monomeric Wastewater

The settling properties and nutrient-removal efficiency were very similar before and after the transition from simple to complex monomeric wastewater, but the bacterial community changed dramatically. The class of Actinobacteria with *Tetrasphaera, Propioniciclava, Propionicimonas*, and *Micropruina*, and the phylum of Saccharibacteria became abundant with the transition from simple to complex monomeric wastewater, while the class of Gammaproteobacteria and in particular *Ca*. Accumulibacter diminished from around 35% to <5%. The genera belonging to Actinobacteria detected with the complex wastewater were previously detected in high abundance in EBPR activated sludge (Kong et al., 2008; Xia et al., 2008; Nielsen J. L. et al., 2012; Nielsen P. H. et al., 2012; Stokholm-Bjerregaard et al., 2017) treating real wastewater. A higher abundance of these bacteria in AGS treating complex wastewater than in AGS treating simple wastewater was therefore expected. In AGS treating complex polymeric wastewater, percentages of <1–33% of Actinobacteria were reported (Liu et al., 2017; Cetin et al., 2018; Kang et al., 2018; Swiatczak and Cydzik-Kwiatkowska, 2018). As in the present study, comparisons between microbial communities of AGS treating simple and complex wastewater detected very few Actinobacteria when VFA were the main carbon source (Holling, 1973; Layer et al., 2019). In particular, Layer et al. (2019) compared the microbial communities of the sludge during four parallel start-up of AGS sequencing batch reactors fed with different wastewater types, two municipal wastewater and two synthetic wastewater, a simple and a complex polymeric. The carbon compositions of the two sythetic wastewater were similar to the simple synthetic wastewater and the complex polymeric wastewater used during the study presented here. In both studies, in the AGS fed with simple wastewater, the guild of PAO was mainly composed of *Ca*. Accumulibacter and the guild of GAO of members of the Competibacteraceae, whereas in the AGS fed with complex wastewater the guild of PAO comprised *Ca*. Accumulibacter and *Tetraspaera* and the guild of GAO comprised members of the Competibacteraceae and *Micropruina*. The two sets of microbial communities differed mainly by the proportions of PAO and GAO. The high concentrations of phosphate (22 mgl^−1^) in the wastewater of this study favored PAO (20–40%) over GAO (5–15%), whereas the concentrations of phosphate in the wastewater of the study of Layer et al. favored GAO (4–20%) over PAO (2–4%).

In this study, these fermenting bacteria were negatively, and for most of them significantly, correlated with the concentration of ${\text{PO}}_{4}^{-}$ in the bulk water at the end of the anaerobic phase. Marques et al. (2017) measured the P release after anaerobic incubation of a sludge containing both *Ca*. Accumulibacter and *Tetrasphaera* with different carbon sources. With acetate or propionate the P release per c-mmol was much higher than with the glucose, aspartate, glutamate or glycine. Therefore, the link between high proportions of these fermenting bacteria, mostly abundant with the complex wastewater, and the low amount of ${\text{PO}}_{4}^{-}$ measured at the end of the anaerobic phase can results from the lower amount of VFA. *Tetrasphaera* has a different metabolism than the classical PAO *Ca*. Accumulibacter which hydrolyzes its polyphosphate reserves to take up VFA in anaerobic conditions. The hypothesis has been raised that fermentation of sugars and amino acids may allow *Tetrasphaera* to generate enough energy to get back anaerobically the released phosphate (Marques et al., 2017). This could explain the lower ${\text{PO}}_{4}^{-}$ concentration measured at the end of the anaerobic phase when the wastewater contains glucose and amino acids.

The members of Saccharibacteria, formerly named Candidate phylum TM7, detected in this study, are only characterized at the phylum level. Their metabolism is therefore unknown. Yet, the metagenome assembled genomes of other members of this phylum (e.g., *Ca*. Saccharimonas) have recently been obtained from EBPR sludge samples (Albertsen et al., 2013). The reconstructed genome of *Ca*. Saccharimonas suggests an obligate fermentative metabolism, taking up glucose and releasing acetate and lactate, with the ability to survive in aerobic conditions through oxidative stress protective enzymes, such as superoxide dismutase and glutathione peroxidase (Albertsen et al., 2013). The fact that the uncharacterized members of Saccharibacteria were abundant in the system only with complex wastewater, suggests that they may also have the ability to ferment glucose or amino acids. The studies of other members of this phylum revealed bacteria with a small genome and parasitic/commensalistic/symbiotic lifestyles (McLean et al., 2018; Bor et al., 2019). Nevertheless, the results presented here show that a high proportion of Actinobacteria and Saccharibacteria is not detrimental to the granulation and the settling properties of the AGS, since the SVI_3_ of the AGS stayed below 50 ml g^−1^ with the complex monomeric wastewater. Moreover, it is likely that the amplitude of this phenomenon is minimized. Indeed, Actinobacteria are difficult to lyze and their abundance is generally underestimated by amplicon-sequencing based evaluations (Albertsen et al., 2015).

The dramatic decrease of the relative abundance of *Ca*. Accumulibacter is difficult to explain. First, there didn't seem to be a limitation in VFA availability. VFA were still present in the influent although at lower concentrations, and the fermentative bacteria produce VFA upon glucose and amino acid fermentation. Indeed, the fermentative PAO *Tetrasphaera* can ferment various sugars and amino acids anaerobically and release succinate, acetate and other fermentation products (Kristiansen et al., 2013; Barnard et al., 2017). The fermentative GAO *Micropruina* also releases the fermented carbon mainly as acetate and lactic acid (McIlroy et al., 2018). *Propioniciclava* and *Propionicimonas* are known to ferment glucose and other carbon source and release propionic acid (Bae et al., 2006; Zhang et al., 2017). Hence, the fermentation products released by these fermentative bacteria should allow classical PAO and GAO to remain in the system. Second, the “disappearance” of *Ca*. Accumulibacter from the AGS was temporary and this PAO was detected again at around 20%, 8 months later. The most likely hypothesis for the sudden decrease of *Ca*. Accumulibacter is an infection by a bacteriophage. Indeed it is common that a dominant population of a bacterial community is attacked by a specific phage (Rodriguez-Brito et al., 2010). More specifically, sudden decreases of *Ca*. Accumulibacter, detected in EBPR activated sludge by using quantitative FISH, were attributed to a lytic *Ca*. Accumulibacter-associated bacteriophage (Barr et al., 2010b). As in the present study, this event was associated with an increase of the proportion of *Ca*. Competibacter and a deterioration of the P-removal efficiency. The phage attack is supported by previous analyses performed on the AGS present in the reactor before the beginning of the experiment (Adler, 2019). The genomic DNA of the phages EBPR podovirus 1 (EPV1) was detected in the DNA sequences of the granules sequenced individually (Leventhal et al., 2018). This phage was previously detected in the metagenomes from an EBPR reactor and was proposed to be able to infect *Ca*. Accumulibacter (Skennerton et al., 2011). A potential phage attack would explain the particular bacterial community composition of stable states 2 and 3.

### 4.4. Transition From Complex Monomeric to Polymeric Wastewater

The changes in the bacterial communities after the transition from complex monomeric to polymeric wastewater were more subtle than the bacterial community changes during the first transition from simple to complex monomeric influent, as shown in the PCoA of the bacterial communities at stable states. Likely some of the bacteria able to ferment sugars and amino acids have also the capability to hydrolyze polymeric compounds (Xia et al., 2008). One of the major changes observed during this transition was the increase of *Zoogloea*. In AGS, this heterotrophic EPS producer (Larsen et al., 2008) is a marker of VFA leakage in the aerobic phase (Weissbrodt et al., 2012). It had already began to thrive after the second pH incident. At that time, the biomass was lower and even if the volume exchange ratio was adapted to the amount of biomass, the COD concentrations at the end of the anaerobic phase were slightly higher than the usual ones. The genus *Niabella, Lysobacter*, and unclassified members of the Saprospiraceae family were detected in significantly higher abundance after the introduction of polymers in the influent wastewater. Members of the genus *Niabella* are able to hydrolyze starch (Weon et al., 2006) or other polymers, such as casein and chitin (Kim et al., 2007). Members of the genus *Lysobacter* have a protease or a starch hydrolyzing activity (Siddiqi and Im, 2016; Chhetri et al., 2019). The Saprospiraceae family is known for its capability to hydrolyze “complex carbon sources” (McIlroy and Nielsen, 2014). These hydrolyzing capabilities may have helped these bacteria to thrive in the reactor fed with complex polymeric wastewater.

The settling properties of the sludge were significantly lower with the polymeric wastewater compared to the monomeric wastewater, in particular because part of the biomass was composed of flocs. This effect of polymeric compounds on the settling properties were previously observed with AGS treating low to medium-strength wastewater containing an important proportion of polymers (typically municipal wastewaters) (de Kreuk et al., 2010; Martins et al., 2011; Pronk et al., 2015b; Wagner et al., 2015; Derlon et al., 2016; Layer et al., 2019). The link between carbon diffusibility and the settling properties of AGS has been studied by Layer et al. (2019) with four reactors run in parallel and fed with different proportions of diffusible carbon. Higher amounts of diffusible carbon in the influent showed to promote granulation and high settling properties; the AGS fed with monomeric wastewater were characterized by big dense granules and a proportion of flocs below 5%, whereas the AGS fed with polymeric wastewater were smaller and contained 20–40% of flocs. This influence of the wastewater type was hypothesized to be due to the diffusibility of the substrate or to the nature of the microbial communities induced by this substrate. The results of the present study confirm this positive impact the carbon diffusibility on the settling properties since the settling properties of AGS decreased when part of the monomers were replaced by their polymeric version. Moreover, it tends to validate the hypothesis of the direct influence of the substrate diffusibility on the settling properties rather than an indirect influence via the microbial community, since the latter did not change significantly during the transition from complex monomeric to complex polymeric wastewater. Layer et al. proposed that the bacteria located far from the surface of the granules have a limited access lower diffusible carbon, their growth is therefore limited and small granules and flocs are favored (Larsen and Harremoes, 1994; Mosquera-Corral et al., 2003). In both studies, the AGS fed with polymeric wastewater contained 20–40% of flocs. These flocs can play a crucial role in the removal of polymeric compounds and therefore be beneficial to the overall functioning of the AGS (Layer et al., 2019). In this study, the SVI_30_ values of 67–92 ml g^−1^ measured after the transition to polymeric wastewater are fully consistent with the SVI_30_ values of AGS treating polymeric wastewater (Derlon et al., 2018; Layer et al., 2019; Zou et al., 2019) and remain lower than standard SVI_30_ values of conventional activated sludge which are between 100 and 150 ml g^−1^ (Xu et al., 2019). With AGS, high amounts of COD at the beginning of the aerobic phase are believed to be responsible for the development of floccular or less dense structures due to the overgrowth of aerobic heterotrophs (Pronk et al., 2015a). Yet, hydrolysis being a slow process (Goel et al., 1998; de Kreuk et al., 2010) and the rate limiting step for the COD uptake by the bacterial communities (Morgenroth et al., 2002), it is likely that part of the COD is not hydrolyzed during anaerobic phases shorter than 2 h. An increased amount of COD available at the beginning of the aerobic phase could be an important factor of the deterioration of the settling properties of the sludge observed with polymeric wastewater (Weissbrodt et al., 2012).

The transition to polymeric wastewater was first accompanied by a progressive replacement of the granules by floccular biomass and no new granule seemed to form during 3 months. Apparition of new granules was detected 2 months after the end of the transition to polymeric wastewater (day 788). Yet, no significant change in the bacterial community was noted around that day, therefore one can only speculate as to the reasons for the temporary loss of granulation. A potential reason resides in biofilm regulation possibly via quorum sensing (Tan et al., 2014) due to lower COD availability for the bacteria involved in biofilm formation. At the time of the transition, the AGS had been fed almost exclusively with monomers during 3.5 years. It is possible that the expression of genes involved in the hydrolysis of polymers had been down-regulated via epigenetic modifications (e.g., DNA methylation) (Sanchez-Romero and Casadesus, 2020) and that it took 3 months for the microbial community to adapt its metabolism to the new composition of the wastewater and start granulating again. Under this hypothesis, the decreased amount of COD available for the bacterial community for several months, caused a stress that induced the later to temporarily invest less in biofilm formation (Burne, 1998; Jefferson, 2004; Jefferson et al., 2004). No significant changes in the COD values were measured at the end of the anaerobic phase after the addition of polymeric compounds in the influent. Nevertheless, our measurements of COD were likely underestimating the real concentrations of polymeric COD at the end of the anaerobic phase. First, the water samples were filtered at 0.45 μm before COD measurements. These filters possibly retained part of the polymeric COD. Second, it is possible that part of the polymers were not detected in the bulk water because they were adsorbed at the surface of the granules and flocs (de Kreuk et al., 2010). In addition, the polymeric COD, not much available to the bacterial community, could have been utilized by the eukaryotic populations of the AGS, which is able to take up macro particles before their hydrolysis (Schwarzenbeck et al., 2004; de Kreuk et al., 2010).

### 4.5. N-Removal According to the Different Wastewater Types

The ammonium-removal was generally close to 100% whatever the wastewater type. It was not the case for N-removal which was visible by the presence of variable concentrations of nitrate in the effluent. In this study, few simultaneous nitrification-denitrification (SND) was noticed during the aerobic phase and the N-removal was around 50%. The full aeration was therefore replaced by intermittent aeration. It extended a little the length of the cycles but improved substantially the N-removal (up to 80%). During the transition from simple to complex monomeric wastewater, N-removal performance was impaired. This coincided with the abrupt decrease of *Ca*. Accumulibacter in the AGS and the drop of P-removal performance. It is likely that most *Ca*. Accumulibacter present in BNR sludge are implicated in denitrification (Kim et al., 2013). Therefore, the sudden decrease of *Ca*. Accumulibacter could be a cause of the impaired denitrification. The total COD which decreased slightly during this transition can be another valid explanation for the lower N-removal. After the transition, the COD of the wastewater was increased to 600 mgO_2_ l^−1^ and both good P- and N-removal were recovered.

Globally, N-removal was slightly lower with the polymeric wastewater in comparison with the monomeric wastewater. Denitrification capabilities are widespread across the bacterial kingdom (Wang et al., 2014). Numerous putative denitrifying bacteria were found in the AGS treating monomeric and polymeric wastewater. In addition, it was reported that AGS with very similar microbial communities can achieve very different N-removal depending on the total COD of the influent (Layer et al., 2019). This shows that the combination of operational conditions and the bacterial community composition acts on the N-removal performance of the AGS, a sufficient COD content being an important factor (Wagner et al., 2015; Wang et al., 2018; Layer et al., 2019). With polymeric wastewater, part of the COD may not be available for the bacteria that participate to the denitrification and are located more in the interior of the granules. The denitrification, which requires COD, is therefore lower than with a wastewater containing only diffusible COD.

### 4.6. The Microbial Communities in Flocs and Granules

A dynamic equilibrium tends to establish between flocs and granules, with the bacteria detaching from the surface of the granules forming new flocs, and parts of broken granules and flocs aggregating or serving as seed for new granules (Barr et al., 2010a; Gonzalez-Gil and Holliger, 2014; Zhou et al., 2014; Szabo et al., 2017). Several studies report big differences between the microbial communities in flocs and granules (Liu et al., 2017), but generally they are more similar between flocs and granules fractions in a single reactor than between the same fractions in different reactors (Zhou et al., 2014; Layer et al., 2019). The data collected during this experiment confirms that even though some taxa were slightly enriched in flocs or in granules, the overall bacterial community was globally similar in the two fractions.

Several factors can favor the enrichment of specific taxa in one or the other fraction. The sludge retention time is higher in granules due to their excellent settling properties (Ju and Zhang, 2015). Therefore, slow growing bacteria can be preferentially enriched in granules. The GAO *Ca*. Competibacter and the fermentative PAO *Tetrasphaera* were maybe more abundant in the granular fraction for this reason. Depending on the stability of the granules, the microbial populations located far from the surface of the granule are more or less exposed to erosion (Szabo et al., 2017). In the case of very resistant granules, those microbial populations will be sparse in the flocs fraction and the flocs will mainly contain bacteria that are located at the surface of the granules (Weissbrodt et al., 2013). Indeed, in the present study aerobic heterotrophic bacteria, such as *Thiothrix, Propionicimonas*, and *Zoogloea* were enriched in flocs. Conversely to other bacteria, the ones having the capability to use nitrate as electron acceptor may have to possibility to grow in the regions located under the surface of the granules. These bacteria will be less susceptible to erosion and therefore enriched in granules. This could also explain why *Rhodobacter, Ca*. Competibacter, and *Tetrasphaera* were generally more abundant in the granules.

## 5. Conclusion

This study was designed to assess separately the impact of the introduction of fermentable and polymeric compounds in the influent wastewater on the bacterial community, the settling properties and the nutrient removal performance of an AGS sequencing batch reactor previously fed with a simple monomeric influent containing acetate and propionate. The high resolution monitoring of the bacterial community of AGS shows that it is very dynamic and that other factors than wastewater composition can trigger important changes in its composition as indicated by multiple stables states with complex monomeric wastewater as influent. The nutrient removal performances of AGS could be globally maintained after the wastewater transitions, and despite different incidents, through the plasticity and the functional redundancy of its bacterial community. In particular, this study confirms that the co-existence of *Tetrasphaera* and *Ca*. Accumulibacter provides a robustness to biological dephosphatation. As in EBPR, the class of Actinobacteria have an important place in the AGS microbial communities treating complex wastewater. This study shows that their high abundance is not detrimental for granulation. Actinobacteria are underrepresented in the AGS fed with a simple wastewater containing VFA as main carbon source. The introduction of polymeric compounds, not readily available for prokaryotes and less accessible for the bacteria located far form the granules surface, had a negative impact on the settling properties and, in a lesser extent, the N-removal of the AGS, without disrupting the composition of the microbial community. After an adaptation period of 2 months, the settling properties of AGS treating complex polymeric wastewater were still lower than those of AGS fed with monomeric wastewater but remain very interesting compared to conventional activated sludge.

## Data Availability Statement

The datasets generated for this study can be found in the European Nucleotide Archive (ENA) under the study accession number ERP122292.

## Author Contributions

AA and CH designed and managed the project. AA analyzed the DNA sequences, performed the statistical analysis, and wrote the manuscript. CH reviewed it and provided valuable edits. All authors contributed to the article and approved the submitted version.

## Conflict of Interest

The authors declare that the research was conducted in the absence of any commercial or financial relationships that could be construed as a potential conflict of interest.

## Footnotes

^1^An interference of amino acids with the compounds of the Spectroquant kit lowering the ammonium concentrations detected was noted on non-diluted samples.

## Supplementary Material

Supplementary Data Sheet 2 Bray-Curtis distance matrix of the bacterial OTU (99%) relative abundance in the AGS of the different samples.

Supplementary Data Sheet 3 P-values of the t-test between the mean relative abundance of the bacterial taxa (at genus level, after Hellinger transformation) in the different stable states. Mean relative abundances of the bacterial taxa in the different stable states.

Supplementary Data Sheet 4 P-values of the t-test between the mean relative abundance of the bacterial taxa (at genus level, after Hellinger transformation) in the different stable states grouped by wastewater type (simple, complex monomeric and complex polymeric). Indication of the divergent, abundant and discriminant bacterial taxa. Mean relative abundances of the bacterial taxa in the different wastewater types at stable state.
