# Supplementary material for: Multistability and Reversibility of Aerobic Granular Sludge Microbial Communities Upon Changes From Simple to Complex Synthetic Wastewater and Back
Source: Front Microbiol. 2020 Nov 26;11:574361. doi: 10.3389/fmicb.2020.574361 (PMC7726351; doi:10.3389/fmicb.2020.574361)
Supplement: Supplementary file 1 [file Data_Sheet_1.pdf]

Supplementary Material

1 SUPPLEMENTARY DATA

1.1 Complement 1 : experiments proceedings

During experiment 1, the composition of the wastewater was progressively changed from simple monomeric to complex monomeric : on day 77, 96 and 109, approximately 20%, 40% and 66% of the COD, respectively, was replaced by glucose and amino acids in equivalent COD proportions. From day 121 onward, the TN concentration of the medium was fixed to 56 $\mathrm{mg\,l^{-1}}$ by adapting the initial ammonium concentraction so that it counterbalance the ammonium produced by amino acids degradation. The reactor was duplicated on day 333 by distributing equally the biomass of RB in the two reactors RA and RB, and they were run in parallel with the complex monomeric wastewater. The influent wastewater lacked trace element solution from day 564 to 582 in RB and from day 578 to day 582 in RA. Following this, the decision was taken to stop RB and restart it with half of the biomass from RA.The composition of the wastewater was then changed from complex monomeric to complex polymeric, in RB : on day 639, 680 and 729, 10%, 20% and 33% of the COD in the form of glucose and amino acids was replaced by starch, a polymer of glucose, and peptone, a mixture of peptides of different length and amino acids ($<$10KD, BD Peptone Bacto, Fisher scientific), in equivalent COD proportions. During experiment 2, the composition of the wastewater was progressively changed from complex monomeric back to simple monomeric, in RA : on day 638, 684, and 732, the proportion of COD in the form of glucose and amino acids was reduced to 40%, 20% and 0%, by replacing it with acetate and propionate.

2 SUPPLEMENTARY TABLES AND FIGURES

2.1 Tables

Table S1. Recipes for the preparation of the different synthetic wastewater used in Experiment 1 and Experiment 2 in g per 10 l.

denomination		simple		transition					complex monomeric		transition		complex polymeric
compound	formula	1	1B	2	2B	3	3B	4A	4B	4C	5	6	7
						C-medium							
Sodium acetate trihydrate	$C_2H_3O_2Na$ *$3H_2O$	42.84	56.70	34.27	45.36	25.70	34.02	14.28	18.90	18.90	18.90	18.90	18.90
Sodium propionate	$C_3H_5O_2Na$	17.52	22.87	14.02	18.30	10.51	13.723	5.84	7.62	7.62	7.62	7.62	7.62
Glucose monohydrate[a]	$C_6H_{12}O_6$ * H_2O	-	-	3.78	5.5	7.55	11.01	12.59	18.38	18.38	15.6	12.84	9.175
Starch[a]	-	-	-	-	-	-	-	-	-	-	4.50	7.51	
Magnesium sulfate heptahydrate	$MgSO_4$ * $7H_2O_2$	1.77	1.77	1.77	1.77	1.77	1.77	1.77	1.77	1.77	1.77	1.77	1.77
Calcium chloride dihydrate	$CaCl_2$ * $2H_2O$	1.65	1.65		1.65		1.65	1.65	1.65	1.65	1.65	1.65	1.65
Potassium chloride	KCl	3.575	3.575	3.575	3.575	3.575	3.575	3.575	3.575	3.575	3.575	3.575	3.575
						NP-medium							
Alanine	$C_3H_7NO_2$	-	-	0.485	0.707	0.970	1.414	1.617	2.360	2.360	2.000	1.650	1.178
Arginine	$C_6H_{14}N_4O_2$	-	-	0.517	0.754	1.035	1.508	1.725	2.510	2.510	2.140	1.760	1.257
Aspartic acid	$C_4H_7NO_4$	-	-	0.725	1.056	1.450	2.113	2.416	3.520	3.520	2.990	2.465	1.761
Glutamic acid	$C_5H_9NO_4$	-	-	0.534	0.778	1.068	1.557	1.781	2.590	2.590	2.210	1.816	1.297
Glycine	$C_2H_5NO_2$	-	-	0.818	1.192	1.635	2.383	2.725	3.970	3.970	3.380	2.780	1.986
Leucine	$C_6H_{13}NO_2$	-	-	0.286	0.416	0.572	0.833	0.953	0.953	1.390	1.180	0.972	0.694
Proline	$C_5H_9NO_2$	-	-	0.342	0.498	0.684	0.997	1.140	1.660	1.660	1.410	1.163	0.831
Peptone		-	-	-	-	-	-	-	-	-	1.90	3.81	6.35
Ammonium chloride	NH_4Cl	18.93	18.93	18.93	16.315	18.93	13.702	18.93	7.91	7.91	7.91	7.91	7.91
Potassium hydrogenophosphate	K_2HPO_4	7.305	7.305	7.305	7.305	7.305	7.305	7.305	7.305	7.305	7.305	7.305	7.305
Monobasic potassium phosphate	KH_2PO_4	2.855	2.855	2.855	2.855	2.855	2.855	2.855	2.855	2.855	2.855	2.855	2.855

[a] Glucose and starch solution was added after the autoclave of the C-medium to prevent glucose degradation.

Table S2. Table of the discriminant taxa of the study. The sign '-' indicates that the taxon was significantly less abundant in the second wastewater type compared to the first one. Conversely, the sign '+' indicates that the taxon was significantly more abundant in the second wastewater type compared to the first one. The average abundances of the taxa according to the three wastewater types are in Supplementary Data Sheet 4

Genus	simple to complex monomeric	simple to complex polymeric	complex monomeric to polymeric
Thiothrix	+	-	-
Ca. Competibacter		-	-
CPB S18			-
CPB C22&F32	+		-
Pseudoxanthomonas			-
Lysobacter		+	+
Hydrogenophaga		+	
Ca. Accumulibacter	-	-	
Rhodocyclus		-	-
Zoogloea	-		+
Dechloromonas			+
B142 (f)	+		-
Rhodobacter	+	+	
Hyphomonadaceae (f)	-	-	+
OPB56 (f)	-	-	
Nitrospira			-
Propionicicalva	+	+	-
Micropruina	+	+	
Propionicimonas	+	+	-
sbr-gs28	+	+	+
Nocardioides	+	+	
Tetrasphaera	+		-
CYCU-0281	-		+
Saprospiraceae		+	+
Niabella	+	+	+
Cytophagaceae (f)	-	-	
Flavobacterium	-	-	-
Saccharibacteria (p)	+	+	+

Table S3. Pearson correlations between discriminant taxa (as defined in section 2.11) and nutrient removal.

taxon	TN_rem	PO4_rem	COD_AN	PO4_AN
Thiothrix	0.332	0.118	-0.122	0.166
Ca. Competibacter	0.124	-0.202	-0.140	0.189
CPB_S18	-0.138	-0.362	-0.057	0.240
CPB_C22&F32	0.290	0.286	-0.090	-0.046
Pseudoxanthomonas	-0.140	-0.129	-0.008	-0.089
Lysobacter	-0.060	0.121	0.126	-0.107
Hydrogenophaga	-0.285	-0.191	-0.035	-0.191
Ca. Accumulibacter	0.070	0.119	-0.109	0.359
Rhodocyclus	-0.025	-0.360	-0.057	0.165
Zoogloea	-0.177	-0.104	0.108	-0.198
Dechloromonas	-0.109	0.177	-0.022	0.003
B142 (f)	0.103	0.002	-0.047	-0.105
Rhodobacter	-0.069	0.044	0.035	-0.244
Hyphomonadaceae (f)	0.131	-0.076	-0.174	0.089
DB1-14 (o)	0.054	-0.093	-0.041	0.257
OPB56 (f)	0.081	-0.010	-0.118	0.004
Nitrospira	-0.146	-0.389	-0.070	0.094
Propioniciclava	-0.015	0.087	0.155	-0.220
Micropruina	-0.118	0.109	0.074	-0.418
Propionicimonas	0.072	0.120	-0.016	-0.257
sbr-gs28	0.123	0.358	0.139	0.169
Nocardioides	-0.008	0.321	0.008	-0.224
Tetrasphaera	-0.093	-0.197	-0.048	-0.108
CYCU-0281	-0.097	-0.016	-0.001	0.002
Saprospiraceae (f)	-0.086	0.140	0.058	0.134
Niabella	-0.034	0.164	0.204	-0.224
Cytophagaceae (f)	0.002	-0.140	-0.092	-0.034
Flavobacterium	0.188	-0.069	-0.115	0.394
Saccharibacteria (p)	-0.081	0.266	0.152	-0.218

Table S4. P-values of Pearson correlations between discriminant taxa (as defined in section 2.11) and nutrient removal.

taxon	TN_rem	PO_rem	COD_AN	PO4_AN
Thiothrix	$5.50{\times}10^{-6}$	$1.16{\times}10^{-1}$	1.03×10^{-1}	2.65×10^{-2}
Ca. Competibacter	9.75×10^{-2}	6.67×10^{-3}	6.18×10^{-2}	1.11×10^{-2}
CPB_S18	6.45×10^{-2}	6.17×10^{-7}	4.49×10^{-1}	1.19×10^{-3}
CPB_C22&F32	8.09×10^{-5}	1.04×10^{-4}	2.32×10^{-1}	5.43×10^{-1}
Pseudoxanthomonas	6.11×10^{-2}	8.59×10^{-2}	9.19×10^{-1}	2.36×10^{-1}
Lysobacter	4.23×10^{-1}	1.07×10^{-1}	9.29×10^{-2}	1.53×10^{-1}
Hydrogenophaga	1.11×10^{-4}	1.02×10^{-2}	6.39×10^{-1}	1.03×10^{-2}
Ca. Accumulibacter	3.51×10^{-1}	1.13×10^{-1}	1.47×10^{-1}	8.07×10^{-7}
Rhodocyclus	7.38×10^{-1}	7.60×10^{-7}	4.48×10^{-1}	2.75×10^{-2}
Zoogloea	1.76×10^{-2}	1.66×10^{-1}	1.49×10^{-1}	7.95×10^{-3}
Dechloromonas	1.45×10^{-1}	1.80×10^{-2}	7.73×10^{-1}	9.63×10^{-1}
B142 (f)	1.71×10^{-1}	9.75×10^{-1}	5.32×10^{-1}	1.62×10^{-1}
Rhodobacter	3.57×10^{-1}	5.59×10^{-1}	6.40×10^{-1}	9.90×10^{-4}
Hyphomonadaceae (f)	8.14×10^{-2}	3.12×10^{-1}	1.99×10^{-2}	2.39×10^{-1}
DB1-14 (o)	4.73×10^{-1}	2.14×10^{-1}	5.84×10^{-1}	5.16×10^{-4}
OPB56 (f)	2.79×10^{-1}	8.90×10^{-1}	1.15×10^{-1}	9.53×10^{-1}
Nitrospira	5.08×10^{-2}	7.21×10^{-8}	3.54×10^{-1}	2.12×10^{-1}
Propioniciclava	8.39×10^{-1}	2.49×10^{-1}	3.84×10^{-2}	3.08×10^{-3}
Micropruina	1.17×10^{-1}	1.47×10^{-1}	3.22×10^{-1}	5.70×10^{-9}
Propionicimonas	3.40×10^{-1}	1.08×10^{-1}	8.27×10^{-1}	5.13×10^{-4}
sbr-gs28	9.98×10^{-2}	8.96×10^{-7}	6.43×10^{-2}	2.39×10^{-2}
Nocardioides	9.13×10^{-1}	1.20×10^{-5}	9.14×10^{-1}	2.58×10^{-3}
Tetrasphaera	2.16×10^{-1}	8.27×10^{-3}	5.19×10^{-1}	1.51×10^{-1}
CYCU-0281	1.99×10^{-1}	8.33×10^{-1}	9.86×10^{-1}	9.77×10^{-1}
Saprospiraceae (f)	2.54×10^{-1}	6.18×10^{-2}	4.37×10^{-1}	7.38×10^{-2}
Niabella	6.49×10^{-1}	2.82×10^{-2}	6.08×10^{-3}	2.54×10^{-3}
Cytophagaceae (f)	9.75×10^{-1}	6.15×10^{-2}	2.20×10^{-1}	6.48×10^{-1}
Flavobacterium	1.19×10^{-2}	3.59×10^{-1}	1.25×10^{-1}	4.99×10^{-8}
Saccharibacteria (p)	2.81×10^{-1}	3.13×10^{-4}	4.21×10^{-2}	3.39×10^{-3}

2.2 Figures

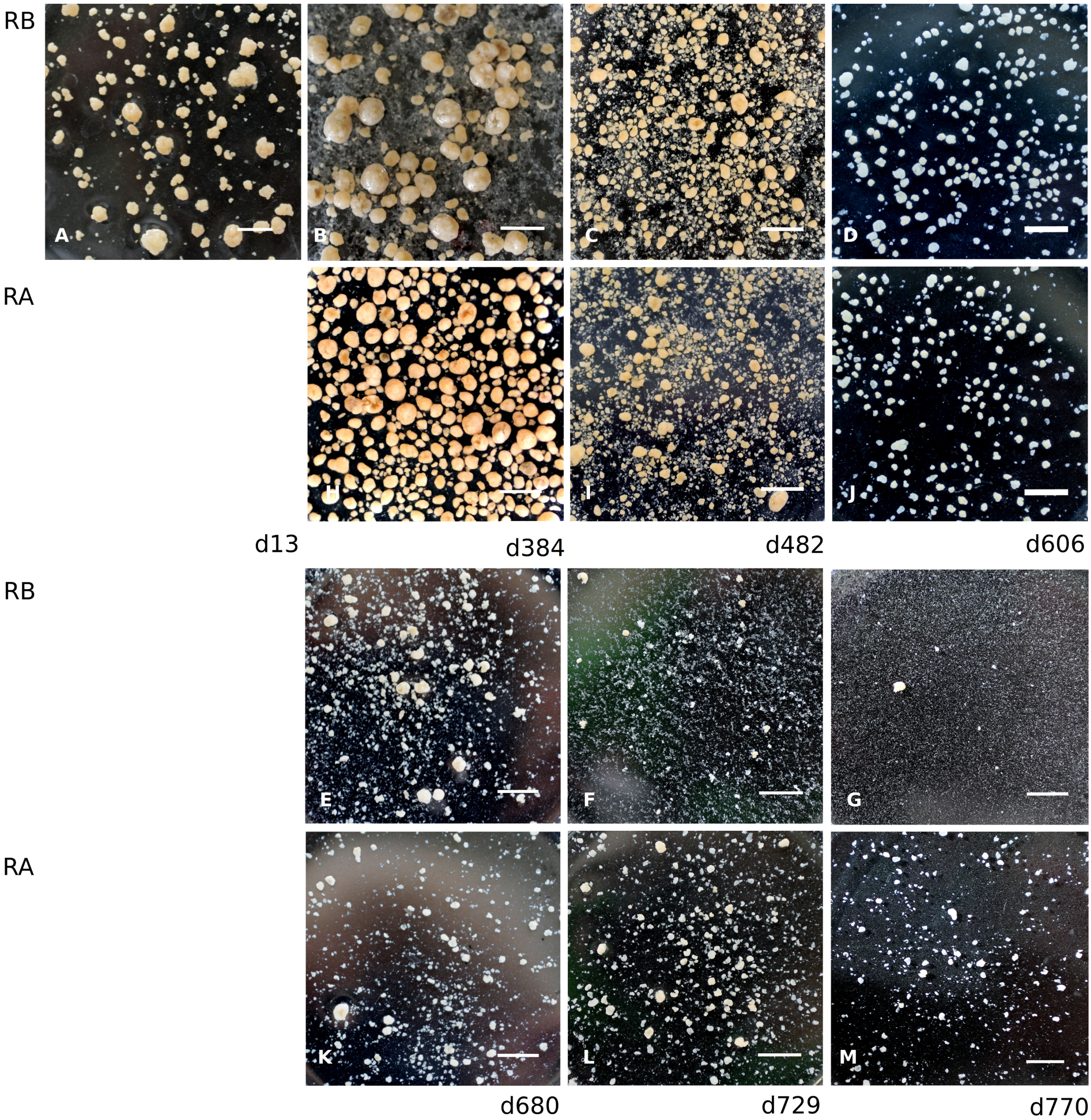

Figure S1. Pictures of the biomass sampled during experiment 1 and experiment 2. Pictures of the biomass sampled in RB are on the first line (A-G) and the biomass sampled in RA is on the second line (H to M). The white bars represent 1 cm. The first column corresponds to biomass sampled on day 13 with medium 1 (simple wastewater). The three following columns corresponds to days 384, 482 and 606, when the reactors RA and RB were operated with medium 4 (complex monomeric wastewater). On the three last columns are the pictures of the biomass collected during the transition to polymeric wastewater (RB, days 680, 729 and 770) and the transition back to simple wastewater (RA, days 680, 729 and 770).

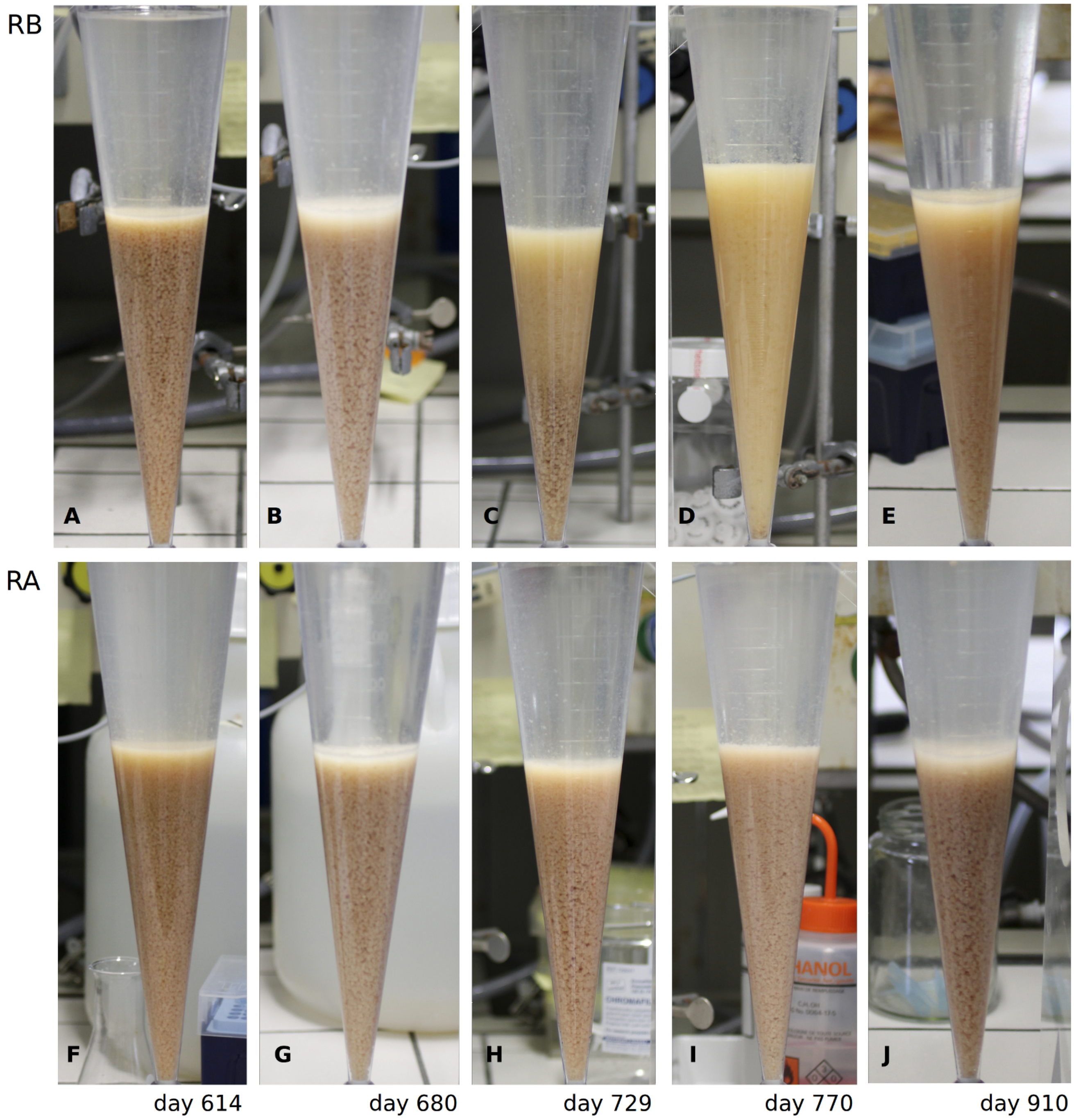

Figure S2. Pictures taken after 10 minutes of settling, with the exception of the picture of day 770 which was taken after 3 minutes of settling, with biomass borrowed form reactor RB (A-E) and RA (F-J) during SVI measurements.

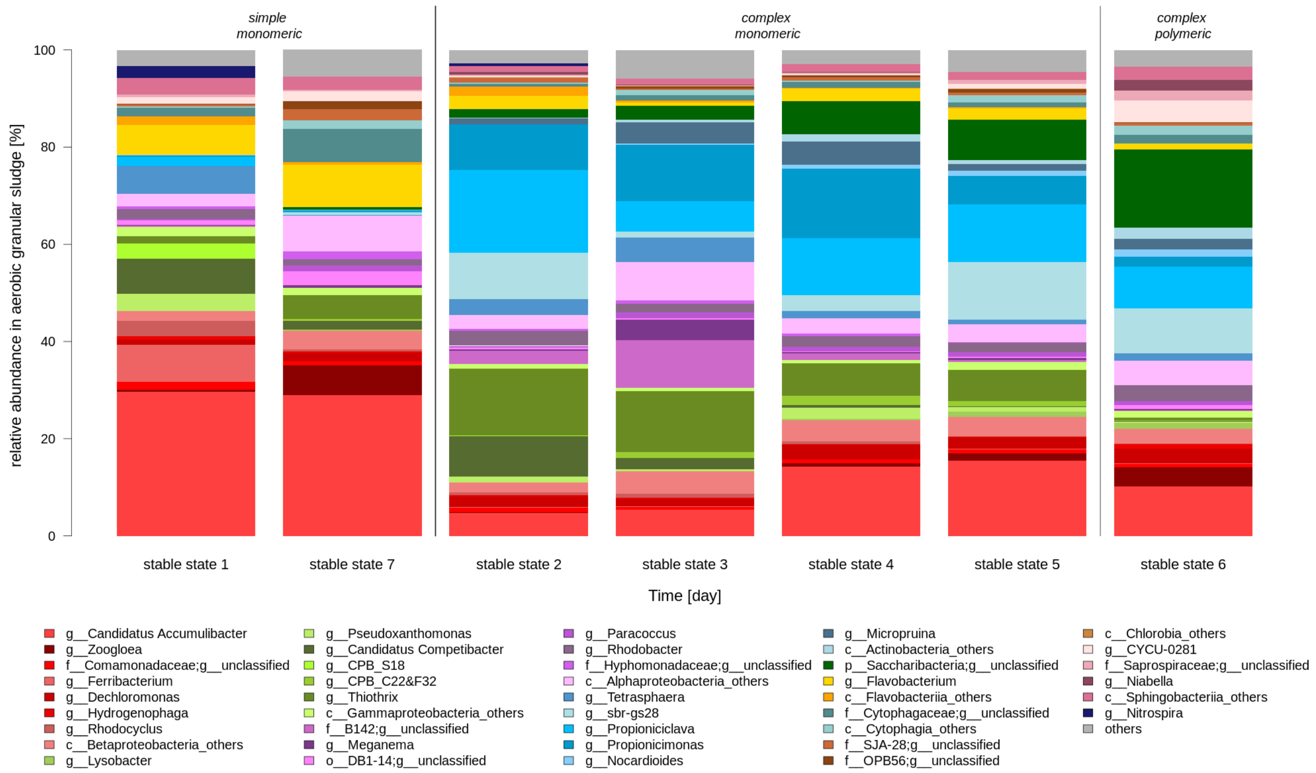

Figure S3. Average composition of the bacterial community in the different stable states. The genera are colored according to the class they belong to, with the exception of the Betaproteobacteriales, previously the class of Betaproteobacteria, colored in red. They were recently merged with the Gammaproteobacteria (Parks et al.,2018), here colored in light green.

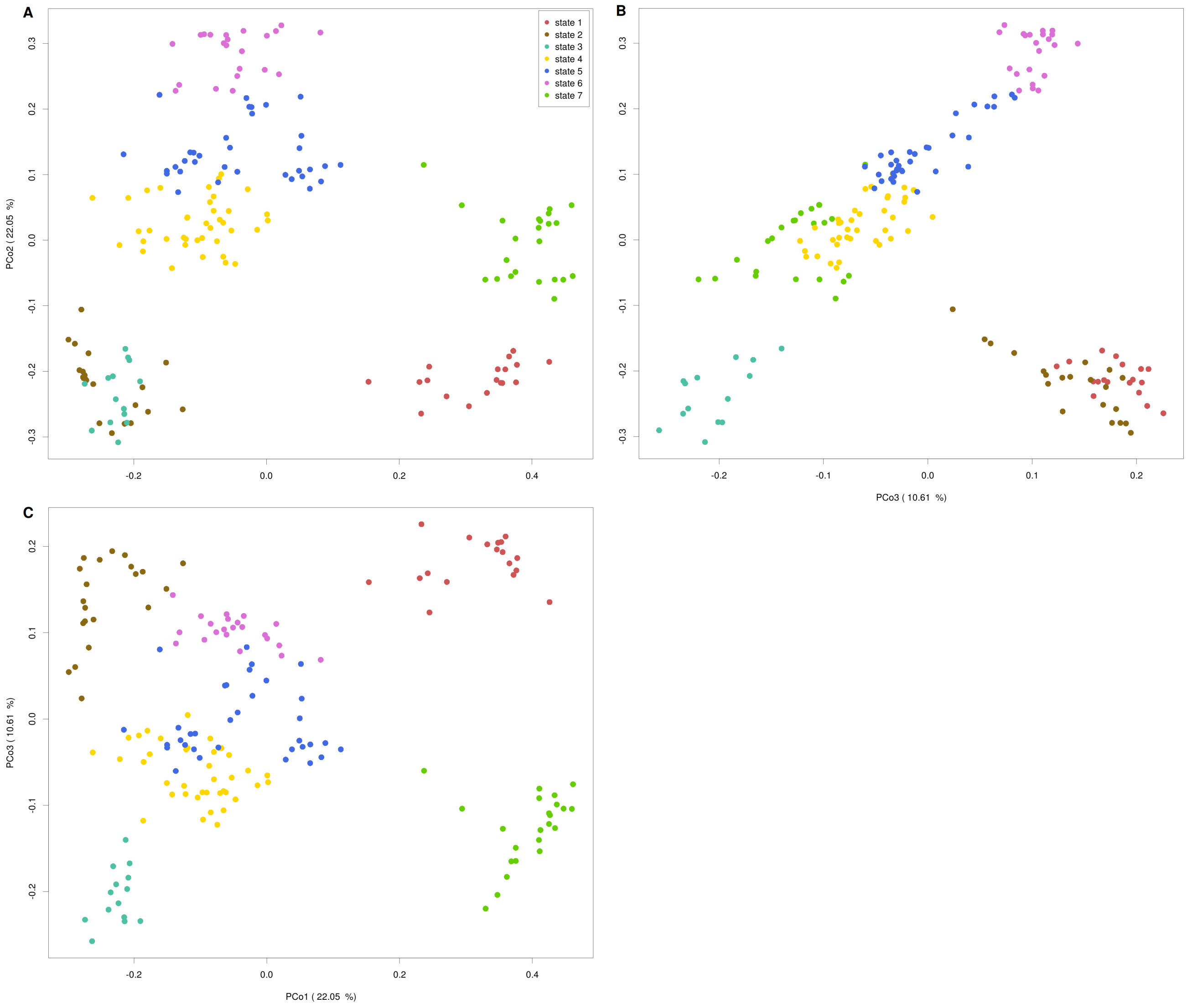

Figure S4. Principal coordinate analysis of the Bray-Curtis distance of the bacterial communities of the stable states of the two experiments on the 1st and 2nd axis (A), on the 3rd and 2nd axis (B) and on the 1st and the 3rd axis (C).

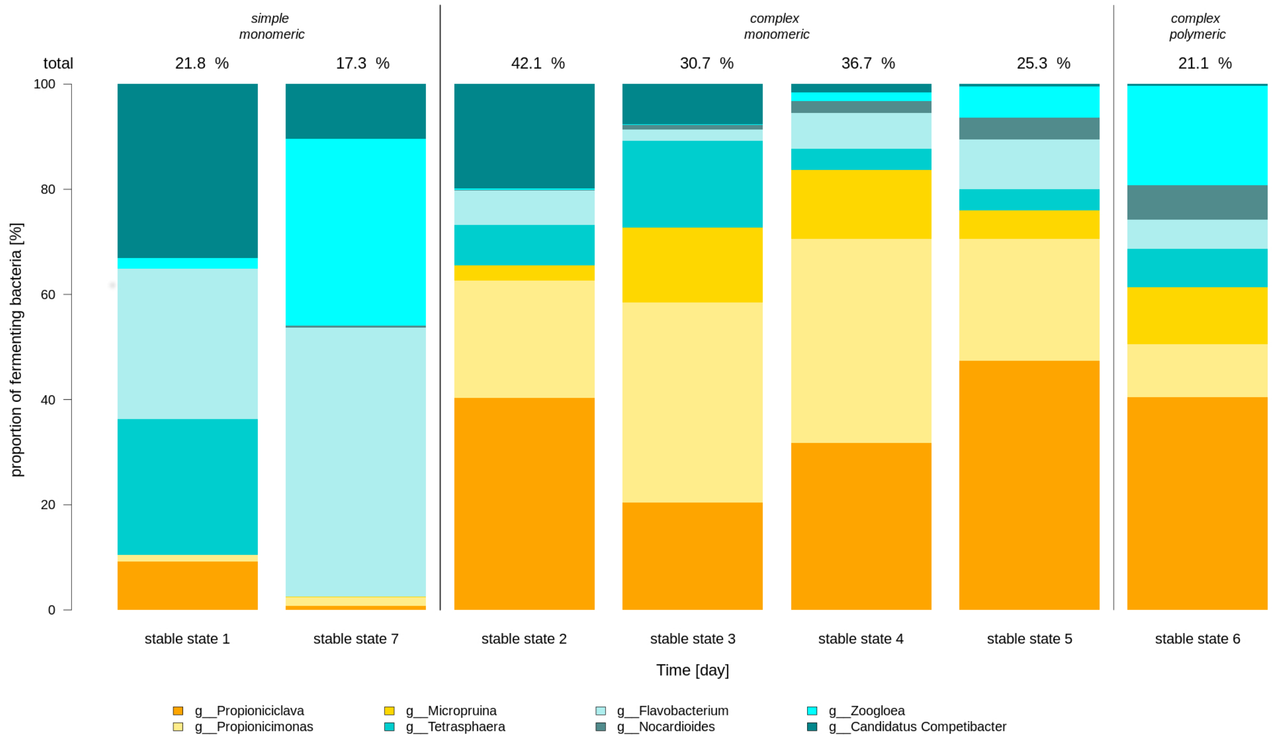

Figure S5. Composition of the guild of bacterial genus with a fermenting metabolism provided by MiDAS database (McIlroy et al., 2015), in the different stable states. The genus for which the fermentative function was detected for all the members (positive) are shown in yellow, the genus for which the fermentative function was detected for part of the members (variable) are shown in blue. The total proportions of fermenting and putatively fermenting genera in the different stable states are shown at the top of the barplot.

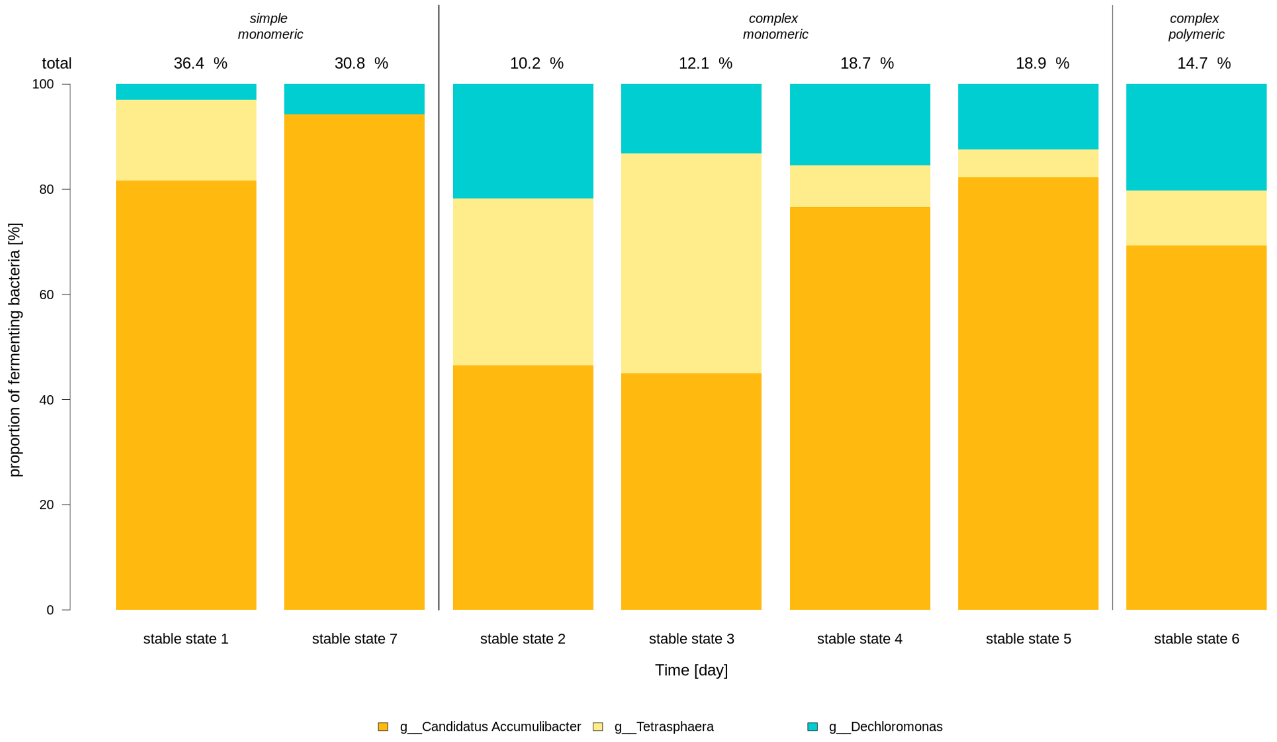

Figure S6. Composition of the guild of bacterial genus with a PAO metabolism provided by MiDAS MiDAS database (McIlroy et al., 2015), in the different stable states. The genus for which the PAO function was detected for all the members (positive) are shown in yellow, the genus for which the PAO function was detected for part of the members (variable) are shown in blue. The total proportions of PAO and putative PAO genera in the different stable states are shown at the top of the barplot.

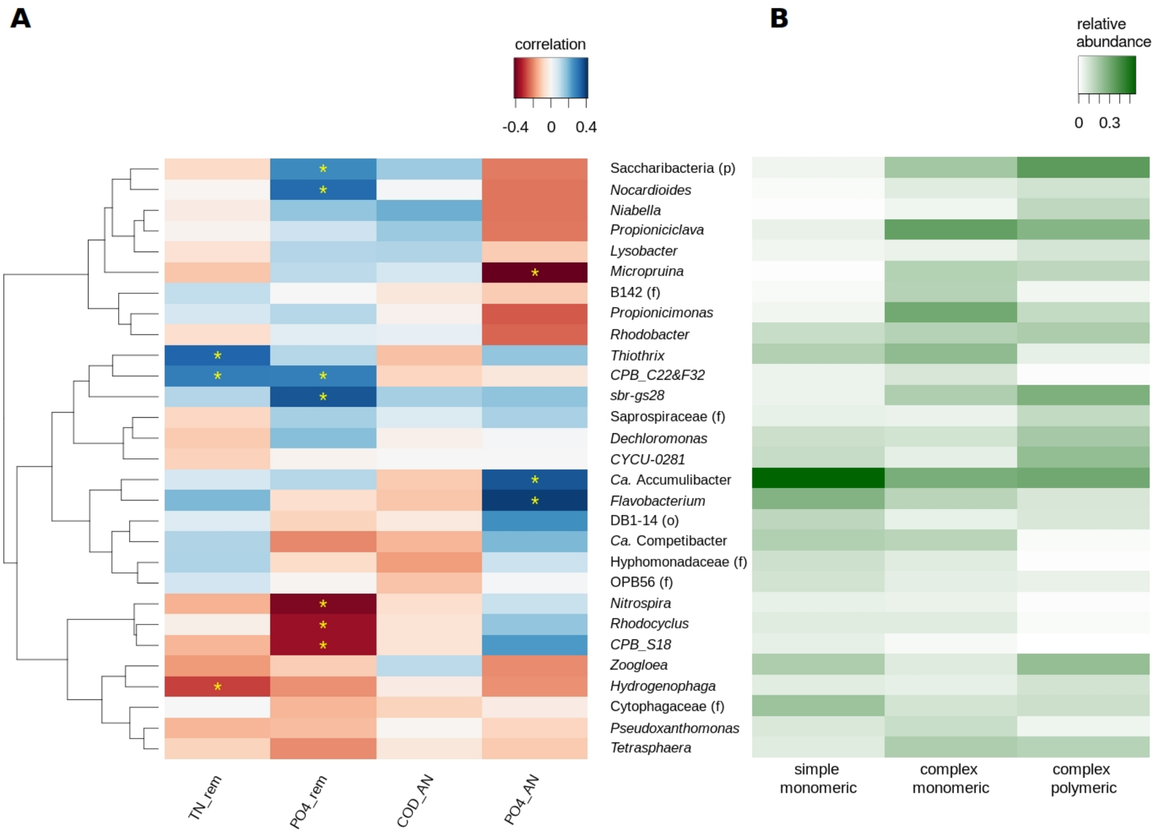

Figure S7. Correlation (pearson) heatmap between the discriminant taxa and the nutrient removal during experiment 1 and 2 (A) and average Hellinger-transformed relative abundance of these taxa in the stable states corresponding to the different wastewater types (B).

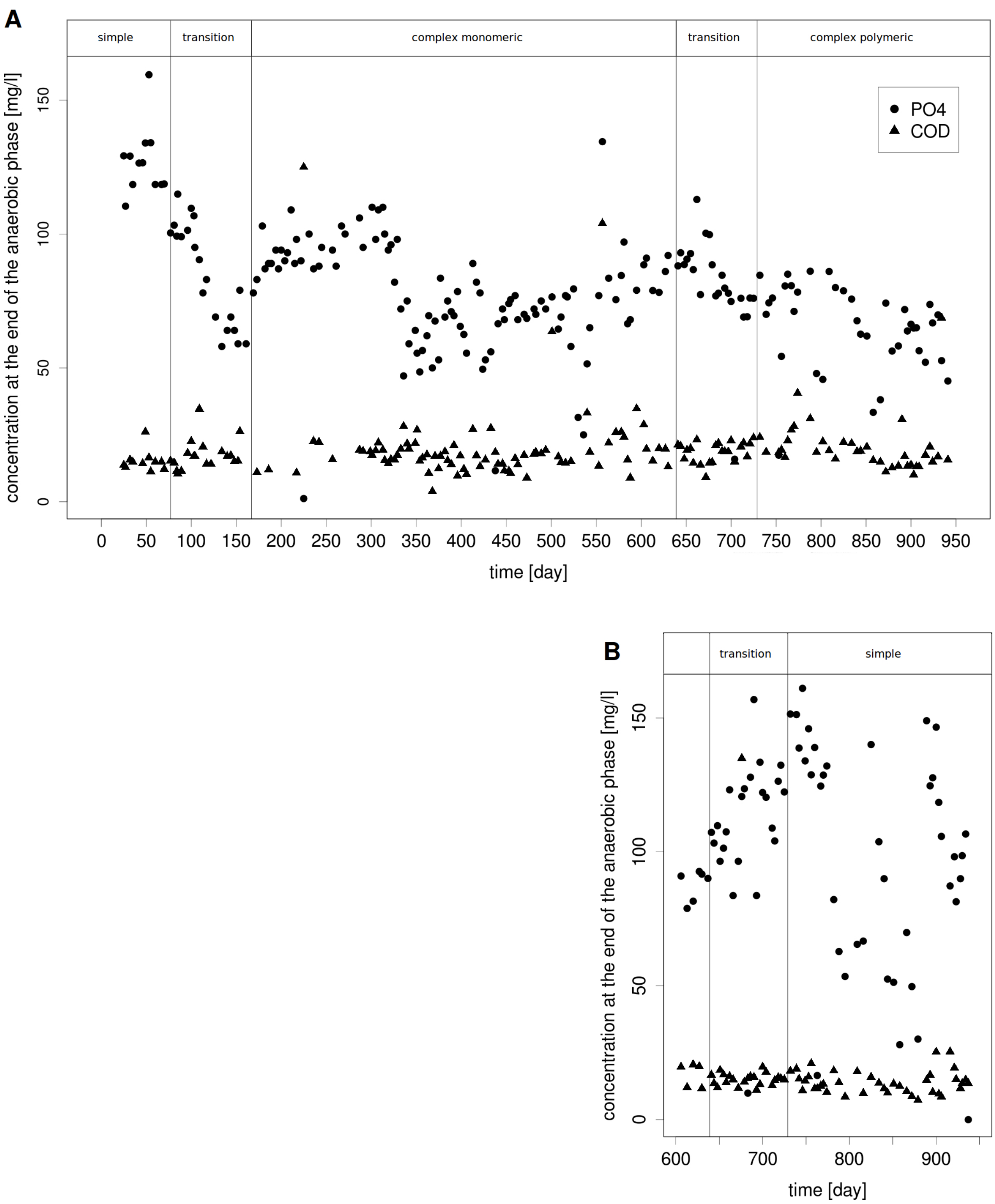

Figure S8. PO_4^- and COD concentrations in the bulk water measured at the end of the anaerobic phase (A) during experiment 1 and (B) experiment 2.
